# Evaluating the Structural Response of Amphiphilic Monolayers to Environmental Stimuli

**DOI:** 10.1021/acs.langmuir.5c03473

**Published:** 2025-09-05

**Authors:** Allison V. Cordova-Huaman, Nicholas C. Craven, Marea J. Blake, Benjamin Doughty, Clare McCabe, G. Kane Jennings

**Affiliations:** † Department of Chemical and Biomolecular Engineering, 5718 Vanderbilt University, Nashville, Tennessee 37235-1604, United States; ‡ Interdisciplinary Materials Science Program, Vanderbilt University, Nashville, Tennessee 37235-1604, United States; § Chemical Sciences Division, 6146 Oak Ridge National Laboratory, Oak Ridge, Tennessee 37831, United States; ∥ School of Engineering and Physical Sciences, 3120 Heriot-Watt University, Edinburgh, Scotland EH14 4AS, U.K.

## Abstract

Amphiphilic monolayers composed of end groups with distinct polar and nonpolar functional groups offer rapid and reversible interfacial adaptation in response to environmental stimuli such as a change in interfacial medium polarity. We have synthesized and characterized a suite of monolayers with functional groups of competing polarity designed to reconfigure their interfacial chemical composition in response to solvent polarity. In these films, the end group is designed to be able to reorient and expose the functional groups that minimize the interfacial free energy between the film and the environment. Using a combination of spectroscopic, computational, and wettability studies, we have investigated the responsive interfacial behavior of different end groups upon exposure to environments with varying polarities. Contact angle measurements across a series of polar and dispersive probe liquids reveal trends that reflect the underlying molecular flexibility and composition. Vibrational sum frequency generation (SFG) spectroscopy and atomistic molecular dynamics (MD) simulations confirm solvent-driven reorientation of the end groups, with restructuring observed at the interface. To quantify these effects, we have developed a surface energy calculation model that incorporates solvent-induced surface rearrangements into the estimations. Our findings reveal a strong dependence of surface energy and switching behavior on the length and flexibility of the functionalities in the end group, which affects the exposure of certain interfacial compositions under different solvents. These results offer new insights into the design of adaptive monolayers and provide a framework for evaluating solvent-responsive surfaces.

## Graphical abstract

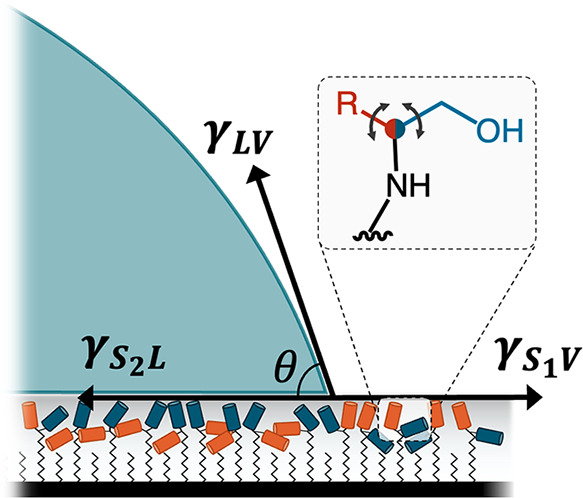

## Introduction

The ability to fabricate amphiphilic coatings that impart responsive properties to various surfaces is essential for a wide range of environmental and clinical applications. Solvent-responsive films, in particular, have been extensively utilized in areas such as sensing, oil–water separation, and antifouling coatings for biological implants, membranes, and microfluidic devices.^1–5^ These coatings are characterized by their ability to reversibly alter the organization of their functional groups (i.e., segments, side chains, or end groups) to different interfacial conditions. The observed response is governed by the difference in surface energies of the functionalities, entropic contributions from molecular flexibility, and specific intermolecular interactions among the segments.^6^ Among these factors, surface energies provide an attractive strategy for driving molecular rearrangement due to the tendency of a system to minimize its interfacial energy with the environment.

Amphiphilic copolymers are a class of materials widely used as responsive coatings, composed of both hydrophobic and hydrophilic segments. Their composition, with functionalities of competing polarity, enables switching surface rearrangement in response to environmental cues through macromolecular reorganization.^6,7^ Such cues can include changes in the contacting medium—e.g., from air to saturated water vapor or from a nonpolar solvent like toluene to a polar solvent like water. These environmental transitions prompt the segregation of nonpolar (i.e., hydrophobic) or polar (i.e., hydrophilic) functionalities, thereby minimizing the interfacial energy under varying conditions.

Rearrangement of interfacial composition can also be achieved using amphiphilic monolayers, which offer greater control over the distribution of surface functional groups and more rapid responses than polymers.^8–12^ This makes monolayers particularly suitable for applications requiring fast and adaptive surface behavior. Motivated by the potential of these systems, we synthesized a series of amphiphilic monolayers bearing end groups with competing polarity to investigate their interfacial responsiveness across different solvent conditions. The polarity mismatch between functional groups within the monolayer drives surface reorganization when the interfacial medium shifts from air or a nonpolar solvent to a polar one, promoting exposure of polar groups and thus reducing interfacial free energy. These monolayers are synthesized using adsorbates of different lengths, with the longer ones bearing the functional termini with the competing groups. This design feature provides free volume near the interface, allowing the end group to rapidly reorient the functionalities and enabling fast responses to changes in the contacting medium (Figure 1).

**Figure 1:**
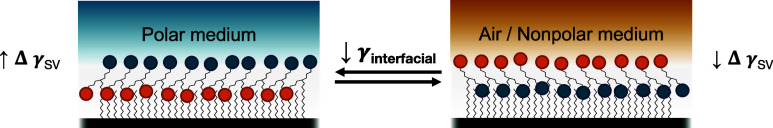
Schematic of the reorientation of the terminal groups of a mixed monolayer to expose either a hydrophilic or hydrophobic interfacial molecular composition upon contact of the film with a polar or air/nonpolar medium, in order to minimize the interfacial free energy (↓γ_interfacial_). Blue and orange circles represent polar and nonpolar functional groups, respectively. The surface becomes enriched with polar functionalities (blue circles) to minimize γ_interfacial_ with polar media, and as a result, the apparent γ_*SV*_ also increases. On the contrary, an increase in nonpolar functionalities (orange circles) at the interface decreases the overall γ_*SV*_ value of the film.

The rapid reorientation and structural reorganization of responsive films such as these amphiphilic films can be studied *in situ* through a range of techniques, including polarization modulation infrared reflection absorption spectroscopy (PM-IRRAS), atomic force microscopy (AFM), and vibrational sum-frequency generation (SFG) spectroscopy.^13–15^ Among these, SFG is particularly informative, as it probes conformational changes directly at the interface, providing insights into film-solvent interactions and the orientation of monolayer end groups under different conditions. Complementary information can be obtained from molecular dynamics (MD) simulations, which can reveal both the structure and mobility of the end groups.^16^ We have recently combined atomistic MD simulations and experimental measurements to show that solvent polarity strongly influences surface reorganization, and even slight modifications in the film composition can lead to distinct variations in group exposure and solvent compatibilities.^17^

To quantitatively assess the responsive behavior of these systems, an ability to determine how the interfacial free energy changes in different solvent environments is critical. Furthermore, the determination of the surface energy can reveal the suitability of these materials for different potential applications. However, since surface energy cannot be directly measured, indirect techniques such as contact angle measurements, atomic force spectroscopy (AFS), MD simulations, Hansen solubility parameters, and solid surface deformation have been employed.^18–23^ Among these, contact angle and AFS methods have been extensively utilized to probe the surface energy of materials. Specifically, contact angle measurements analyzed with models like the Owens-Wendt approach are experimentally simpler than AFS while still providing comparable surface energy measurements, as shown by other works on monolayers and polymer thin films.^24–26^

To facilitate the rapid and effective assessment of the surface energy of responsive monolayers, we have developed a model that extends the Owens-Wendt approach to account for environmentally induced surface rearrangements. This model integrates solvent-responsive changes into surface energy estimates derived from contact angle measurements, allowing for a more precise evaluation of changes in surface energy. The responsive characteristics of the responsive monolayers considered in this model are supported by MD simulations and further validated by experimental observations of the interfacial composition using vibrational SFG spectroscopy. In combination, these approaches enhance our ability to quantify the responsive nature of the monolayers, which also can be easily applied to other smart surfaces, providing a framework for the rapid assessment of other adaptive interfacial systems.

## Materials and Methods

### Theoretical Foundation

In contact angle methods, the surface energy of a material is calculated using the air (*V*)—liquid (*L*)—solid (*S*) three-phase contact angle (θ) with different probe liquids. On a smooth surface with no deformations in the normal direction, θ values relate to the surface energy under air (γ_*SV*_), the interfacial free energy between the surface and the probe liquid (γ_*SL*_), and the surface tension of the probe liquid (γ_*LV*_) through Young’s equation.^27^

1 $$\gamma_{\mathrm{SV}}=\gamma_{\mathrm{LV}}\cos\theta +\gamma_{\mathrm{SL}}$$

Young’s equation is derived from a force balance at the air–liquid–solid triple-phase boundary line, not considering the chemical aspects of interfacial interactions. Furthermore, eq 1 cannot be solved by itself, as γ_*SV*_ and γ_*SL*_ are typically unknown. Therefore, several theoretical models have been developed that consider the solid–liquid and liquid–vapor interactions that contribute to the macroscopic contact angle behavior and that help solve for the unknown γ values.^28–32^ The Owens-Wendt equation is the most commonly used method, which considers a geometric-mean correlation between the solid–liquid interfacial interactions that provides further insight into surface properties.^29^

2 $$\gamma_{\mathrm{SL}}=\gamma_{\mathrm{SV}}+\gamma_{\mathrm{LV}}-2\sqrt{\gamma_{\mathrm{SV}}^{D}\gamma_{\mathrm{LV}}^{D}}-2\sqrt{\gamma_{\mathrm{SV}}^{P}\gamma_{\mathrm{LV}}^{P}}$$

In eq 2, γ_*SL*_ results from dispersive and polar interactions between the surface (γ_*SV*_^*D*^ and γ_*SV*_^*P*^) and the probe liquid (γ_*LV*_^*D*^ and γ_*LV*_^*P*^). Combining eqs 1 and 2 yields eq 3, which can be expressed in a linearized form for direct determination of γ_*SV*_^*D*^ and γ_*SV*_^*P*^ by plotting the left-hand side versus $\sqrt{\gamma_{\mathrm{LV}}^{P}/\gamma_{\mathrm{LV}}^{D}}$ .

3 $$\frac{\gamma_{\mathrm{LV}}\left(\cos\theta +1\right)}{2\left(\sqrt{\gamma_{\mathrm{LV}}^{D}}\right)}=\sqrt{\frac{\gamma_{\mathrm{LV}}^{P}}{\gamma_{\mathrm{LV}}^{D}}}\sqrt{\gamma_{\mathrm{SV}}^{P}}+\sqrt{\gamma_{\mathrm{SV}}^{D}}$$

Considering that γ_*SV*_ = γ_*SV*_^*D*^ + γ_*SV*_^*P*^, several authors have used eq 3 to estimate values for both static and responsive surfaces.^33,34^ However, this approach does not account for the fact that responsive surfaces reorganize their interfacial composition when exposed to different probe liquids. Other works have shown that these responsive coatings experience segregation of the functional groups at the interface to minimize the interfacial free energy of the contacting area between the surface and different media (i.e., minimize γ_*SL*_ with liquid media or γ_*SV*_ with air).^6,33–36^ For example, during a water contact angle measurement, a multicomponent surface may switch its interfacial composition inside the three-phase water/solid/air contact line, exposing more polar groups compared to the region under air. This switching is not observed with dispersive probe liquids.^37^ As a result of this superficial reconstruction, two areas with different compositions and surface energies are formed: *S*_1_, corresponding to the surface under air or a dispersive liquid, and *S*_2_, corresponding to the switched surface under polar media (Figure 2). Based on this assumption, we have developed a model derived from the Owens-Wendt equation that can be used for responsive surfaces and considers the surface energy state under polar and dispersive probe liquids.

**Figure 2:**
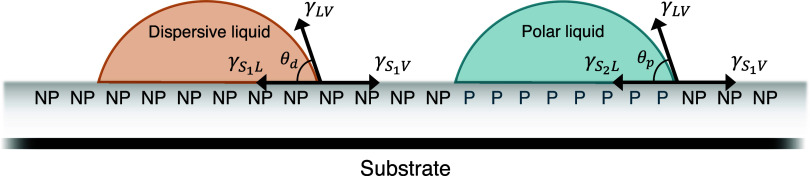
Schematic showing the relationship between three interfacial energies and the sessile contact angle on a dispersive (θ*_d_*, left) and a polar (θ*_p_*, right) liquid drop on a solid surface. *S*_1_ and *S*_2_ represent the surface areas with an enhanced nonpolar (NP) and polar (P) composition when exposed to air or a dispersive liquid and a polar liquid, respectively.

In this model, we first consider a flat and smooth solid surface initially in equilibrium under air with surface energy denoted by γ_*S*_1_ *V*_. Upon exposure to a dispersive liquid, as during contact angle measurements, the surface chemical composition maintains its mostly nonpolar character, maintaining its surface energy γ_*S*_1_ *V*_.^37,38^ However, when the surface is exposed to a polar probe liquid, the interfacial polar chemical composition is enhanced to minimize the interfacial energy (γ_*S*_2_ *L*_) with the probing liquid. Upon reaching equilibrium (i.e., contact angle remains constant), the surface energy varies from the initial γ_*S*_1_ *V*_ value to a new γ_*S*_2_ *V*_ value that relates to the surface composition under the polar liquid and the γ_*S*_2_ *L*_ value. In films where rearrangement occurs only through reorientation of terminal groups, such as amphiphilic monolayers or polymers with both hydrophilic and hydrophobic units in the end groups, this equilibrium is reached almost instantaneously. Other films, such as block copolymers, may require longer times to reach this equilibrium state. However, the time dependence of this surface reorganization lies beyond the scope of this work and has been addressed elsewhere.^39–41^

Considering this variation in surface energies, Young’s equation can be reformulated for dispersive (eq 4) and polar (eq 5) probe liquids using the contact angle measured with each type of probe liquid θ_*d*_ and θ_*p*_, respectively.

4 $$\gamma_{S_{1}V}=\gamma_{\mathrm{LV}}\cos\theta_{d}+\gamma_{S_{1}L}$$

5 $$\gamma_{S_{1}V}=\gamma_{\mathrm{LV}}\cos\theta_{p}+\gamma_{S_{2}L}$$

Similar to the considerations in the Owens-Wendt theory, the interfacial energy of the contacting area between the surface and the dispersive and polar probe liquids (γ_*S*_1_ *L*_ and γ_*S*_2_ *L*_) can be expressed in terms of these components.

6 $$\gamma_{S_{1}L}=\gamma_{S_{1}V}+\gamma_{\mathrm{LV}}-2\sqrt{\gamma_{S_{1}V}^{D}\gamma_{\mathrm{LV}}^{D}}$$

7 $$\gamma_{S_{2}L}=\gamma_{S_{2}V}+\gamma_{\mathrm{LV}}-2\sqrt{\gamma_{S_{2}V}^{D}\gamma_{\mathrm{LV}}^{D}}-2\sqrt{\gamma_{S_{2}V}^{P}\gamma_{\mathrm{LV}}^{P}}$$

Combining eqs 4 and 6, and eqs 5 and 7, the interfacial free energy can be described as

8 $$\gamma_{\mathrm{LV}}\left(1+\cos\theta_{d}\right)=2\sqrt{\gamma_{S_{1}V}^{D}\cdot \gamma_{\mathrm{LV}}^{D}}$$

9 $$\gamma_{\mathrm{LV}}\left(1+\cos\theta_{p}\right)=\Delta \gamma_{S_{2}V-S_{1}V}+2\left(\sqrt{\gamma_{S_{2}V}^{P}\cdot \gamma_{\mathrm{LV}}^{P}}+\sqrt{\gamma_{S_{2}V}^{D}\cdot \gamma_{\mathrm{LV}}^{D}}\right)$$

Here, Δγ_*S*_2_ *V*–*S*_1_ *V*_ = γ_*S*_1_ *V*_ – γ_*S*_2_ *V*_ represents the difference in the interfacial free energy, and thus the surface composition, between the region under air (*S*_1_) and that under the polar liquid (*S*_2_). This parameter provides a quantitative, energy-based metric to assess functional group switchability in polar liquids versus air, where negative values denote a greater switching response. Plotting the left side of eq 8 versus $2\sqrt{\gamma_{\mathrm{LV}}^{D}}$ yields a linear expression that aids in determining the dispersive energy component γ_*S*_1_ *V*_^*D*^. If we consider that dispersive energy components of films composed mostly of polar or nonpolar groups are similar in value, then we can assume that γ_*S*_1_ *V*_^*D*^ ≈ γ_*S*_2_ *V*_^*D*^.^29,42^ By substituting this calculated dispersive energy component into eq 9, one can solve for the remaining polar energy component, γ_*S*_2_ *V*_^*P*^, and determine the overall surface energy of these films.

The final linearized equations of eqs 8 and 9 for dispersive and polar probe liquids can be expressed as eqs 10 and 11, respectively.

10 $$\gamma_{\mathrm{LV}}\left(1+\cos\theta_{d}\right)=2\sqrt{\gamma_{\mathrm{LV}}^{D}}\cdot \sqrt{\gamma_{S_{2}V}^{D}}$$

11 $$\gamma_{\mathrm{LV}}\left(1+\cos\theta_{p}\right)-2\sqrt{\gamma_{S_{2}V}^{D}\cdot \gamma_{\mathrm{LV}}^{D}}=\Delta \gamma_{S_{2}V-S_{1}V}+2\sqrt{\gamma_{\mathrm{LV}}^{P}}\cdot \sqrt{\gamma_{S_{2}V}^{P}}$$

### Reagents and Materials

10-Undecenyltrichlorosilane (10-UTS, 97%) and *n*-octyltrichlorosilane (OTS, 97%) were purchased from Gelest. 11-Mercapto-1-undecanol (97%) and 1-octadecanethiol (98%) were purchased from Sigma-Aldrich. Anhydrous toluene (99.8%), anhydrous tetrahydrofuran (THF, 99.8%), dimethyl sulfoxide (DMSO, 99.9%), ethylene glycol (EG, >99%), thiodiglycol (TG, >99%), glycerol (G, >99%), and 1,3-diiodopropane (DIP, >99%) were purchased from Sigma-Aldrich. Hydrogen peroxide (H_2_O_2_, 30%), sulfuric acid (H_2_SO_4_, >95%), hydrochloric acid (HCl, 36.5–38.0% w/w), potassium permanganate (KMnO_4_, >99%), sodium periodate (NaIO_4_, >99.8%), thionyl chloride (SOCl_2_, >99%), α-bromonaphthalene (αBN, 97%), and diiodomethane (DIM, >99%) were purchased from Thermo Fisher. Potassium carbonate (K_2_CO_3_, >99%) was purchased from Alfa Aesar. Sodium bisulfite (NaHSO_3_, 97%) was obtained from Acros Organics. Ethanolamine (98%), (*S*)-(+)-2-amino-1-propanol (98%), 1-amino-2-methyl-2-propanol (95%), d-l-2-amino-3-methyl-1-butanol (97%), and (*R*)-(−)-2-amino-1-hexanol (97%) were purchased from Sigma-Aldrich. (±)-2-Amino-1-butanol (97%) and d-l-2-Amino-1-pentanol (98%) were purchased from Thermo Fisher and Santa Cruz Biotechnology, respectively. All reagents and solvents were used as received from commercial suppliers. Single-side polished, boron-doped p-type silicon wafers ⟨100⟩ were purchased from University Wafer (1–10 Ω·cm) and Pure Wafer (0.01–0.02 Ω·cm). Chromium-coated tungsten rods were obtained from R.D. Mathis. Gold shot (99.9%) was purchased from J&J Materials. Quartz slides with dimensions 25.4 × 25.4 × 0.15–0.25 mm^3^ used for SFG characterization were purchased from Thermo Fisher.

### Preparation of Si/SiO_2_ Substrates

Silicon wafers ⟨100⟩ (1–10 Ω·cm) were sequentially rinsed with ethanol, water, and ethanol, and dried in a stream of N_2_, followed by sonication in ethanol for 30 min to displace any remaining contaminants. The substrates were then rinsed with ethanol and dried with a stream of N_2_. The substrates were then dipped into a piranha solution (H_2_SO_4_/H_2_O_2_, 7:3, v/v) for 30 min to hydroxylate the silicon oxide surface, washed by immersion in water, rinsed sequentially with water and ethanol, and then dried thoroughly with N_2_ before starting the silanization process.

### Preparation of Au Substrates

Au-coated substrates were prepared by sequentially evaporating chromium (100 Å) and gold (1250 Å) onto silicon wafers ⟨100⟩ (1–10 Ω·cm).^43^ Depositions were performed in a diffusion-pumped chamber at a rate ≤2 Å·s^–1^ with a base pressure of 4 × 10^–6^ Torr. Wafers were stored at ambient conditions and were rinsed with ethanol and then dried under a stream of N_2_ before being used.

### Synthesis of Single-Component Monolayers

Au-coated substrates were placed in 1 mM ethanolic solutions of 1-octadecanethiol and 11-mercapto-1-undecanol for 24 h to form CH_3_- and OH-terminated monolayers, respectively. After this time, the samples were removed from the solution and rinsed with copious amounts of ethanol and dried in a stream of N_2_.

### Synthesis of Functionalized Amphiphilic Monolayers

Functionalized monolayers featuring amide-linked end groups composed of a polar and a nonpolar competing group were synthesized via a sequential surface reaction approach, as described in previous work.^17^ In short, piranha-treated substrates were immersed in a toluene solution with a 1:1 molar ratio of 10-UTS:OTS with a final concentration of 1 mM of the silane precursors. As shown in previous studies, the resulting mixed monolayers are expected to maintain a similar 1:1 ratio of vinyl- and methyl-terminated adsorbates, with these two components homogeneously mixed.^44,45^ After 3 h, the samples were removed from the solution, rinsed in toluene, water, and ethanol, and dried in a N_2_ stream. The resulting vinyl- and methyl-terminated surfaces were then exposed to a freshly prepared oxidizing solution (KMnO_4_, 0.5 mM; NaIO_4_, 19.5 mM; and K_2_CO_3_, 1.8 mM, pH 6.5) for 24 h to convert the vinyl end group to a carboxylic acid.^46^ After this time, the samples were rinsed in NaHSO_3_ (0.3 M), water, 0.1 N HCl, water, and ethanol.

To prepare for functionalization, the mixed monolayers containing methyl and carboxyl functionalities were immersed in a 5 mM SOCl_2_ THF solution for 24 h to form an acyl chloride-rich surface. Subsequently, the samples were rinsed with THF and immediately placed in a 5 mM DMSO solution of the functional amine. After 24 h, the functionalized samples were removed from the solution, rinsed with DMSO, water, and ethanol, dried in a stream of N_2_, and stored in a capped glass vial.

### Contact Angle Measurements

Contact angle goniometry was used to characterize the wetting properties of the monolayer films, measuring sessile contact angle with a Ramé-Hart manual goniometer. Seven probe liquids, including polar (water, ethylene glycol, thiodiglycol, and glycerol) and dispersive (diiodomethane, diiodopropane, and α-bromonaphthalene) liquids, were utilized. The surface tension components and ratios for these probe liquids are listed in Table 1. A minimum of three measurements were performed on at least two replicates of each film. All contact angle values in this work were recorded immediately after placing the probe liquid droplet on the samples. These values stayed unchanged throughout the measurement period, which was about 10 min for each sample, and are included in Table S1.

**Table 1:** Polar and Dispersive Surface Tension Components of the Probe Liquids^28,36,47–49^

probe liquid (symbol)	γ_*LV*_ (mJ/m^2^)	γ_*LV*_^*P*^ (mJ/m^2^)	γ_*LV*_^*D*^ (mJ/m^2^)	γ_*LV*_^*P*^/γ_*LV*_^*D*^
water (W)	72.8	51	21.8	2.3
glycerol (G)	64	34	30	1.1
ethylene glycol (EG)	48	19	29	0.7
thiodiglycol (TG)	54	15.5	38.5	0.4
diiodomethane (DIM)	50.8	0	50.8	0
diiodopropane (DIP)	46.5	0	46.5	0
α-bromonaphthalene (αBN)	43.9	0	43.9	0

### Attenuated Total Reflectance Fourier Transform Infrared (ATR-FTIR) Spectroscopy

Infrared spectra were employed to determine the chemical composition of the films after each synthetic step. ATR-FTIR was conducted using a Thermo Nicolet 6700 FT-IR spectrometer equipped with a Smart iTR ATR attachment with a diamond crystal plate. Each spectrum was accumulated over 512 scans with a spectral resolution of 2 cm^–1^. Infrared spectra of the functionalized films were taken using porous silicon (PSi) as a substrate. PSi was fabricated by anodic etching of a p-type silicon wafer (0.01–0.02 Ω·cm) in a 15% HF solution in ethanol. A sacrificial layer of PSi was first etched to ensure large pore openings at the surface using a current density of 100 mA/cm^2^ for 100 s and then removed with a NaOH solution (1:9, 1 M NaOH­(aq) to ethanol). The porous layer was then formed using identical conditions to the sacrificial layer.^50,51^ Before their use, PSi films were thermally oxidized at 800 °C for 1 h in a Thermolyne Type 48000 Furnace. Before silanization, piranha-treated PSi substrates were rinsed only with ethanol before the drying step with N_2_. Silanization and functionalization of the PSi substrates were performed following the procedure outlined earlier. A freshly piranha-treated PSi substrate was used as the background for each spectrum.

### Vibrational Sum Frequency Generation (SFG) Spectroscopy

SFG measurements at the quartz/monolayer interface were performed with an instrument described in detail previously.^52^ Briefly, collinear broadband mid-infrared (IR) and narrowband near-IR beams were incident at ∼60° normal to a quartz coverslip surface (thickness ∼0.15-0.25 mm).^53,54^ The beams traveled through the quartz to focus on the buried interface that was suspended over a piranha-cleaned Teflon dish with a cylindrical reservoir. The monolayer-bound surface was positioned to face toward the reservoir, which was left empty for film/air measurements and filled with ∼1 mL of pH 6.4 ultrapure water for film/water measurements. Measurements were made in the SSP combinations where the S-, S-, and P-polarization designations are for SFG, NIR, and IR light sources, respectively. Single frames were collected with an exposure time of 180 s.

The broadband IR light source was centered around 3000 cm^–1^ to probe the CH stretching region. The collected data were background-subtracted and scaled to the nonresonant response from a gold film. For film/water measurements, spectra from IR centered at 3000, 3150, and 3300 cm^–1^ were stitched together using a previously established procedure.^55^ Briefly, individual gold and sample spectra collected at the IR centers listed above were background-subtracted, interpolated to share a common frequency axis, truncated at 1% of the maximum intensity, and then summed together. The resulting data spectra were then divided by the summed gold reference spectra. The intensity of the measured SFG signals (*I*_SFG_) was fit to eq 12

12 $$I_{\mathrm{SFG}}\propto {\left|\sum_{q}\frac{A_{q}}{\omega_{\mathrm{IR}}-\omega_{q}+i\Gamma_{q}}+\chi_{\mathrm{NR}}^{\left(2\right)}e^{i\phi }\right|}^{2}$$

where *A*_q_, ω_q_, and Γ_q_ are the amplitude, frequency, and line width of the *q* ^th^ vibrational mode. The nonresonant background and phase angle are given by χ_NR_^(2)^ and ϕ, respectively.^56,57^ Fitting results are given in Tables S2–S6 in the SI.

### MD Simulation

Molecular simulations were performed on model monolayer films, exposed to the seven solvents and to vacuum. Following the procedure of Craven et al., the MoSDeF simulation tools are used to initialize and parametrize the amorphous silica surface and coatings.^17,58^ A 5 × 5 nm^2^ slab of amorphous silica was generated and then coated with 100 chains randomly across the surface, with half the chains being functionalized with the hydroxyethyl polar group and varying the chemistry of the nonpolar group, and the other half corresponding to the methyl-terminated backfill chains. The OPLS-AA force field in Foyer was used to obtain the model parameters. The bottom 1.8 nm of the silica surface was held fixed.^59–61^ The silica-film interaction force field was obtained from Black et al., which was developed to mimic the synthetic conditions of a silicon wafer treated with piranha solution, using a reactive ReaxFF force field.^62^ A 5 nm slab of solvent was placed on top of the surface. A second film was then generated, inverted, and placed above the top of the solvent to form a sandwich structure.

NVT simulations were performed in GROMACS version 2022 at 298 K with a 10-chain Nose-Hoover thermostat.^63^ An initial three-stage relaxation was performed using the steepest descent energy minimization with the initial energy step distances of 0.001, 0.01, and 0.1 nm at each stage. Equilibration of the film and solvent was then performed with a step size of 0.5 fs for 1 ns. In order to approach ambient conditions between the slabs, the top and bottom films were compressed together at a rate of 2 nm/fs for 1 ns, until a set distance was reached using a Newton–Raphson method to approximate the error until the solvent reached its bulk equilibrium density. A 0.25 ns equilibration step was run until the density calculation was performed again on the solvent between the slabs, and this two-stage compression and equilibration approach was repeated until the bulk density fell within 1% of the expected bulk density between the slabs. Finally, the time step was increased to 2 fs, and an annealing step was performed to raise the temperature up to 358 K and back down to 298 K over 0.5 ns, which helped the chains to overcome any final energy barriers. The production stage was run for 10 ns at 298 K, and sampling was performed for the last 8 ns of the production run. Each simulation was run in triplicate, which resulted in data for six random surface configurations.

The simulation data analysis was performed using MDAnalysis to evaluate the film hydrogen bonds and the end group solvation shell density, ρ_group_, where group refers to either the oxygen atom of the polar group or the last carbon of the methyl-terminated nonpolar group.^64,65^ For the film functionalized with –H as the nonpolar group, the solvation shell analysis of the nonpolar group was made using the hydrogen atom on the pivot carbon. The film hydrogen bonds were calculated using the MDAnalysis hydrogen bonds analysis module, where the acceptors are nitrogen and oxygen atoms in the amide bonds and the polar groups, and the donors are the same atoms with hydrogens. The values of 0.12 nm donor-hydrogen cutoff, 0.3 nm donor–acceptor cutoff, and 120 degrees donor-hydrogen-acceptor angle were used to identify a hydrogen bond. Hydrogen bonds between the film and solvent were found following the same methodology, using the oxygen atoms of the four polar solvents studied. All the reported data include means across 200 frames over 8 ns of simulation time. ρ_group_ has been previously used to compare the switchability of different terminal functionalities and distinguish the relative interfacial contribution of the polar and nonpolar groups.^17^ The method uses a 0.5 nm shell around each chain atom to count the number of solvent atoms, reported as an atom density. For example, under water, the solvation shell density of a polar group (ρ_*P*_) would show the number of water oxygens and hydrogens that fall within 0.5 nm of each polar group oxygen atom, with higher numbers indicating increased interaction between water and a polar group.

## Results and Discussion

### Design and Synthesis of Functionalized Monolayers

To fabricate monolayers with a well-defined amphiphilic surface composition, we employ a bottom-up synthetic strategy based on a sequence of rapid, surface-confined reactions that proceed to near-completion (Figure 3). This approach begins with the assembly of a mixed silane monolayer with methyl and vinyl end groups, the latter of which are then oxidized and activated to form an acyl chloride-rich surface (Figure 3a). These interfacial acyl chloride groups serve as anchoring sites for subsequent functionalization with bifunctional amines. Amines have been selected due to their fast and complete reaction with acyl chloride groups in comparison to other functional groups, such as alcohols.^66^ The functional molecules selected possess a polar group (−OH) and a nonpolar alkyl group of tunable length and structure (e.g., -methyl, -isopropyl, etc.). The nonpolar segment is varied in length and/or shape while the polar functional group maintains a constant hydroxyethyl end (Figure 3b). The polarity mismatch between these functional groups is a significant design criterion, as it enables the fabrication of surfaces with distinct amphiphilic character and therefore with varying degrees of responsiveness. Here, the use of high-yield reactions, such as the amide coupling of the end group, ensures a high degree of functionalization.

**Figure 3:**
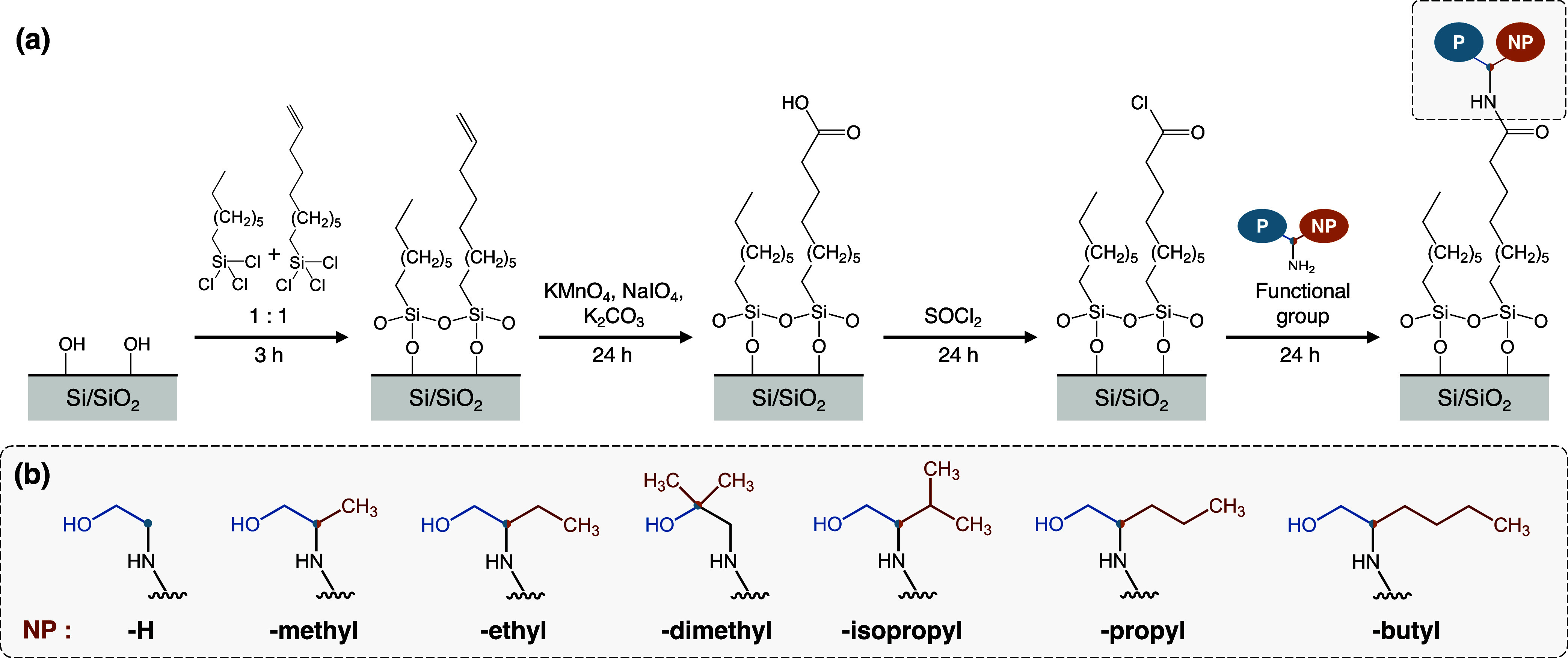
(a) Synthesis scheme for functionalized monolayers via sequential surface reactions on a vinyl-terminated precursor. Details and conditions are further discussed in the Methods section. (b) Chemical structure of the amphiphilic end groups studied, and their nomenclature based on the nonpolar functional group (NP). The pivot carbon is located at the junction of the polar and the nonpolar competing group and is depicted here as a colored circle.

Beyond the design of the functional groups, the overall film structure also plays a significant role in switchability. Specifically, free volume around the end group enables conformational rearrangements and reduces steric constraints, both of which are essential to responsive performance.^44,67^ Here, free volume is introduced by coadsorbing shorter, methyl-terminated backfilling adsorbates with longer, vinyl-terminated active precursors, generating a liquid-like superficial layer atop a high-density underlayer.^17,68^

### Characterization of Amphiphilic Monolayers

Infrared spectra were recorded after each reaction step to determine the chemical composition of the films and assess the efficacy of the synthetic approach. Figure 4 shows the characteristic absorption bands due to the main functional groups of the monolayer film. The spectrum of the vinyl-terminated film exhibits characteristic absorption bands attributed to =CH (3081 cm^–1^) and C=C (1642 cm^–1^) stretching, which completely disappear after the oxidation step that converts these vinyl functionalities to carboxylic acid (C=O stretching, 1718 cm^–1^). The carboxylic acids were then activated to form an acyl chloride-rich surface. Upon exposure of the acyl chloride surface to a functional amine bearing a nonpolar -isopropyl group, absorption bands due to the amide (amide I, 1640 cm^–1^; amide II, 1545 cm^–1^) and methyl functional groups (ν_as_ −CH 2969 cm^–1^) appear. These peaks indicate the expected amide covalent bonding of the end group to the film and confirm the feasibility of this approach to functionalize surfaces with amines that exhibit a diverse array of constituents.

**Figure 4:**
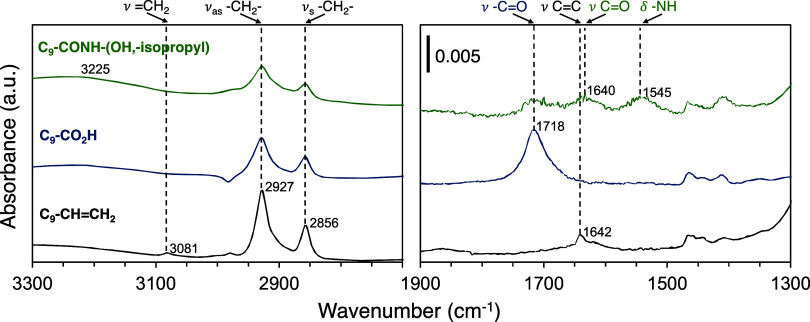
ATR-FTIR spectra of a vinyl-terminated (black), a CO_2_H-terminated (blue), and a 2-amino-3-methyl-1-butanol-modified (green) monolayer showing the sequential reaction steps of the synthesis approach. A porous Si/SiO_2_ substrate was used here to maximize the signal to noise. Spectra have been vertically displaced for clarity. The absorbance scale is the same throughout the functional group region of the IR region.

We employed wettability studies to investigate the changes in surface structure across media of varying polarities by using polar (water, W, and glycerol, G) and dispersive (diiodopropane, DIP, and α-bromonaphthalene, αBN) probe liquids (Table S1 and Figure 5). These probe liquids were selected based on their relatively high surface tension, dominated by polar and dispersive components, respectively. As expected, water contact angles exhibit a trend of decreasing wettability as the nonpolar group increases, reaching a maximum at -ethyl. However, with further elongation of the nonpolar group to -butyl, the water wettability increases, showing an increase in hydrophilicity on these surfaces despite the increased size of the nonpolar group.^17^ This trend is contrary to the observed findings from other studies, where the contact angle values with polar probe liquids tend to rise with increasing hydrophobicity of the end group (i.e., longer nonpolar functional groups).^46^ Contact angle values with glycerol across all functionalized samples portray a similar trend as seen for water, implying a similar surface composition under both polar liquids. Wetting with DIP and αBN exhibit a distinct trend for longer nonpolar groups. For these dispersive probe liquids, the contact angle values increase with nonpolar group size beyond -methyl but are similarly high for the nonpolar groups -dimethyl, -ethyl, -isopropyl, and -butyl, contrary to observations with the polar liquids. Therefore, the wettability with both polar and nonpolar liquids shows a dependence that goes beyond the mere addition of larger dispersive groups to the surface.

**Figure 5:**
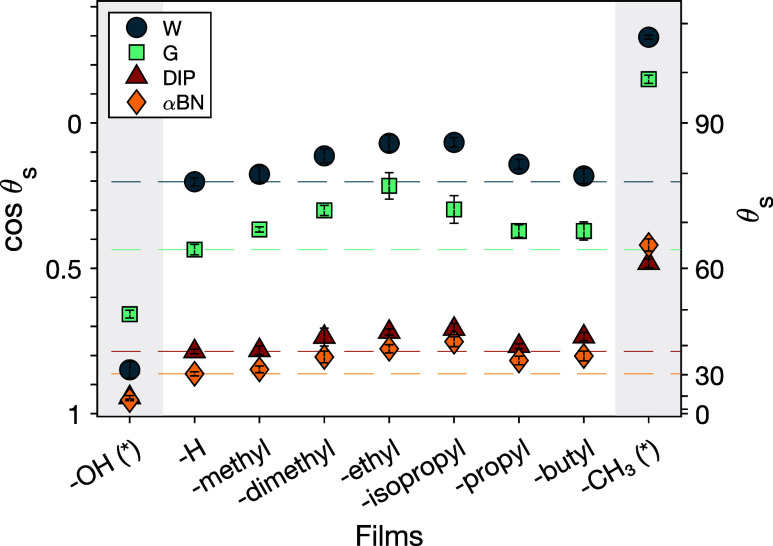
Sessile contact angles for polar and dispersive probe liquids on functionalized amphiphilic monolayers (white section) and purely polar −OH (*) and nonpolar −CH_3_ (*) monolayers (gray section). Amphiphilic monolayers present end groups composed of a polar −OH group and nonpolar alkyl groups of different lengths/structures, which are denoted by their nonpolar group on the *x*-axis. C_11_–OH and C_17_–CH_3_ monolayers on Au-coated substrates were used as surfaces with only polar and nonpolar composition (*), respectively. Water (W) and glycerol (G) were used as polar probe liquids, while 1,3-diiodopropane (DIP) and α-bromonaphthalene (αBN) were used as dispersive probe liquids. Each data point and its error bars represent the mean values of a total of six measurements and the standard deviation, respectively. Horizontal dashed lines show the mean value of the film with −H as the nonpolar group, and these lines are used as a comparison for other surfaces.

In general, these results suggest a molecular-level surface reorganization induced by the polarity change of the contacting probe liquid. In this case, the surface reorganization occurs within the area of the film in contact with the probe liquid, resulting in a surface with a more polar composition under polar liquids and a more dispersive chemical composition under dispersive liquids and air.

### Probing the Interfacial Composition Using SFG

Vibrational SFG spectroscopy, an interface-specific nonlinear optical method, was used to investigate the restructuring of polar and nonpolar groups in the monolayer films when exposed to polar (e.g., water) and nonpolar (e.g., air) environments. Given its inherent selectivity to interfacial species, SFG can elucidate the average ordering of surface-exposed functional groups under different conditions. Figure 6 presents SFG spectra in the SSP polarization combination for monolayers with functional groups bearing -H, -ethyl, and -butyl nonpolar groups (see Figure 2 for structures) upon exposure to air (Figure 6a) and water (Figure 6b). This specific polarization combination and spectral region centered around ∼ 3000 cm^–1^ provide insight into the symmetric CH stretching modes, such as −CH_2_− and −CH_3_ groups.

**Figure 6:**
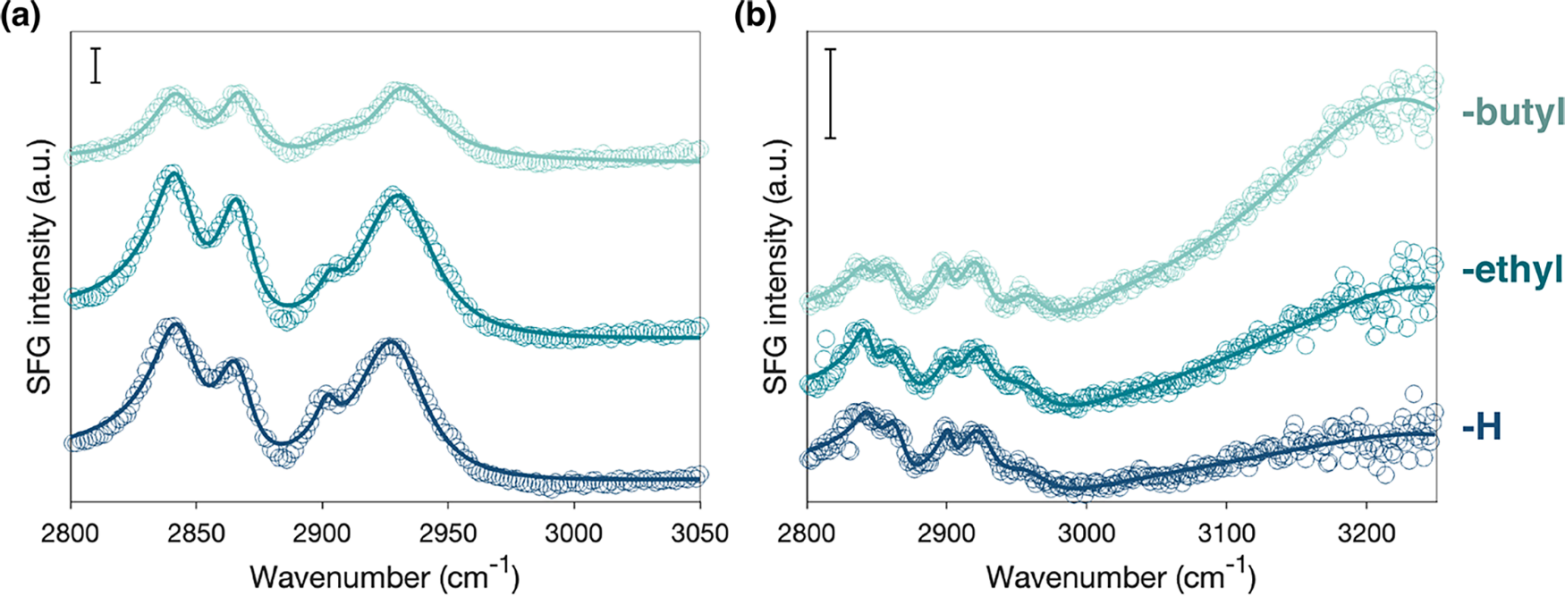
SFG spectra collected at SSP polarization combinations from monolayers functionalized with end functional groups bearing -H, -ethyl, and -butyl as the nonpolar functional group at the (a) film/air and (b) film/water interface. The scale bar shown for each plot equals 0.1 arbitrary units. Spectra are offset for clarity. Data points represent the collected spectra, while the solid lines indicate fits obtained using eq 12 in the Methods section.

Under air, all three SSP spectra exhibit a sharp peak at 2845 cm^–1^, corresponding to the symmetric −CH_2_– stretch (CH_2_–*ss*) from the terminal groups at the film/air interface.^69^ This peak indicates that these alkyl groups dominate the interface across all samples. The feature at 2867 cm^–1^ is attributed to the CH_3_-*ss* resonances from the backfilling chains, with minor contributions from the functional terminal groups containing end methyl groups, as suggested by the results from films without backfilling molecules (Figure S1). Several broad peaks are observed in the ∼ 2900–2950 cm^–1^ range, which arise from overlapping Fermi resonances and out-of-phase asymmetric −CH_2_– and −CH_3_ stretches.^70,71^ These observations indicate that the backfilling chains are most likely oriented along the surface normal, while the nonpolar functional groups are tilted parallel to the surface plane, exposing −CH_2_– groups toward air.

Upon exposure to water, the SSP spectra (Figure 6b) become more dominated by −OH stretching signals (>3000 cm^–1^) as the length of the nonpolar functional group increases from -H to -butyl. This trend suggests that the nonpolar group becomes increasingly buried within the film, allowing the polar −OH groups to reorient toward the aqueous phase. This reorganization of functional groups promotes the formation of a structured hydrogen-bonding network at the interface, as evidenced by the enhanced −OH signals. Among the tested films, the film functionalized with a -butyl nonpolar group exhibits a higher ordering of −OH terminal groups than the film with an -ethyl nonpolar group, despite possessing a longer nonpolar component. This behavior, consistent with contact angle measurements in Figure 5, suggests that the increased conformational flexibility allows this -butyl group to effectively bury within the monolayer, thereby maximizing the exposure of polar functionalities to water. In contrast, the monolayer functionalized with -H, which lacks a nonpolar component, displays a relatively weak −OH stretch signal. The absence of a competing nonpolar group grants the −OH groups greater rotational freedom, likely resulting in a more isotropic orientation at the interface. This reduced orientational ordering leads to lower SFG signal intensity in the −OH stretching region. Thus, while the film functionalized with an -H nonpolar group is inherently more polar, its responsive and less ordered interfacial structure results in weaker spectral features compared to more structurally constrained amphiphilic monolayers.

### Atomistic Insights into Solvent-Induced Monolayer Rearrangement

MD simulations offer further insight into the observed interfacial rearrangements by revealing the molecular-level behavior of terminal groups under different solvent conditions (Figure 7). To assess the extent of polar interactions, the number of interchain hydrogen bonds was quantified when the films were exposed to probe liquids with varying degrees of polarity (Tables S7 and S8). The simulations were performed for monolayers bearing the same chemistries as the synthesized films, and each was equilibrated under vacuum, to reflect the state of the monolayers under air, and under all the probe liquids studied experimentally. Under vacuum and nonpolar solvents such as α-bromonaphthalene, diiodopropane, and diiodomethane, the functionalized monolayers exhibit similar levels of interchain interactions, with an average of 0.75 bonds/chain. In contrast, simulations with polar liquids revealed that less than 0.5 bonds/chain are formed under these media, suggesting significant disruption of the interchain interactions in these environments. Instead, hydrogen bonds are formed strongly between the polar functional groups in the films and the polar solvent molecules, for an average of 0.54, 0.90, 0.77, and 1.06 bonds/chain under thiodiglycol, ethylene glycol, glycerol, and water, respectively (Figure S2 and Table S9). The disruption of interchain hydrogen bond networks is particularly pronounced in the case of water, likely because of its small van der Waals radius and its capacity to fill interfacial voids, reducing interchain hydrogen bonding.

**Figure 7:**
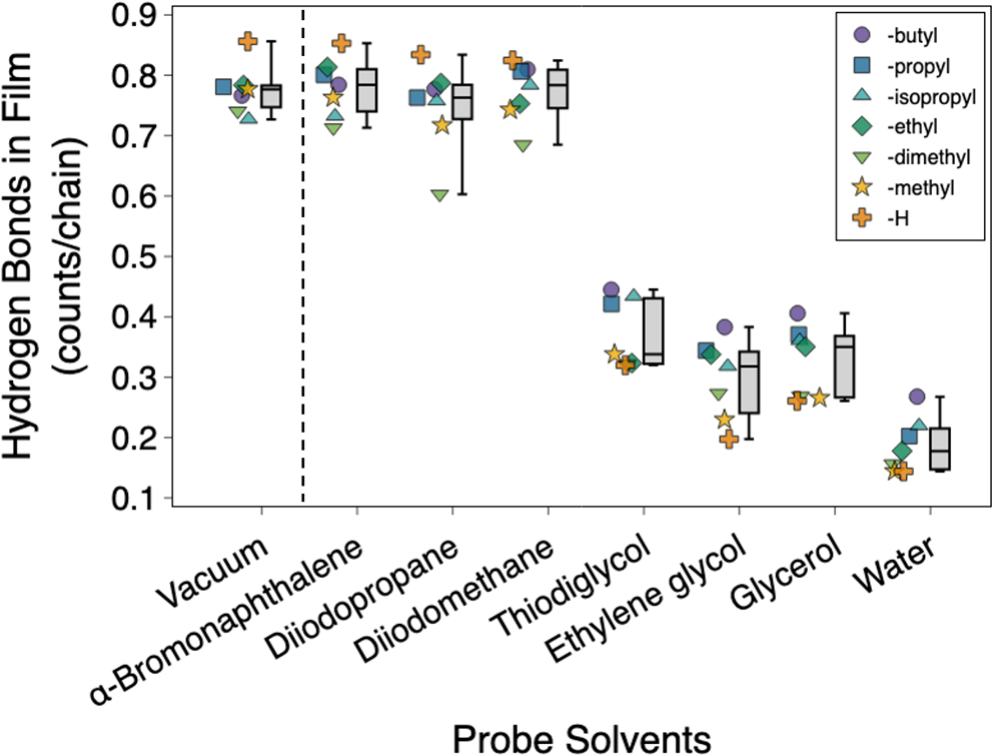
Number of interchain hydrogen bonds in functionalized monolayers simulated under different interfacial media. The dashed vertical line separates data for films in vacuum (e.g., air) from those in nonpolar and polar probe solvents. Hydrogen bond counts are normalized per chain, considering a surface density of 4 chains/nm^2^. For each interfacial medium, individual data points (left) and box-and-whisker plots (right) are shown. Individual data points represent the seven functionalized monolayers, color-coded by their nonpolar functional group. Adjacent box-and-whisker plots summarize the distribution of hydrogen bonds for all films under the same solvent, showing the minimum, 25^th^ percentile, mean, 75^th^ percentile, and maximum values. Solvents are ordered along the *x*-axis in order of increasing polarity (left to right).

The observed differences in hydrogen bond behavior suggest two distinct interfacial states under nonpolar and polar conditions. Under nonpolar conditions, the interface is dominated by interchain hydrogen bonding, while under polar conditions, the solvent competes with the terminal groups and disrupts the interchain interactions. These findings support our surface energy calculation model, which assumes a solvent-dependent reorganization: the interface adopts a more polar or more nonpolar composition depending on the medium. Furthermore, the similarities observed in the state of the monolayers under both dispersive liquids and vacuum are captured, since the model considers that the surfaces have a similar chemical composition under dispersive media and air. The occurrence of this solvent-induced surface rearrangement is also observed in the calculation of the solvation shell for both the polar (ρ_*P*_) and the nonpolar (ρ_*NP*_) functionalities (Figures S3 and S4 and Tables S10–S13). Together with the observed increase in film polarity upon exposure to polar media (Figure S5 and Tables S14 and S15), these results confirm the presence of distinct interfacial states in polar and nonpolar environments, highlighting the solvent-responsive character of amphiphilic monolayers.

The simulated data exhibit consistent and expected switchable behavior across different probe liquids, which aligns with experimental contact angle measurements and SFG results. However, discrepancies emerge when comparing individual films. Specifically, experimental SFG and wetting data indicate increased exposure of the polar −OH group as the nonpolar group extends from -ethyl to -butyl. The intuitive behavior would be the reverse, or that increasing the length of the nonpolar functional group would lessen the exposure of polar functional groups. Both the current simulated ρ_*P*_ values and prior results from Craven et al. show a steady decrease as the nonpolar group increases in length from -ethyl to -butyl.^17^ Hydrogen bonding analysis (Figures 7 and S2) shows a similar trend, with the film with -butyl as the nonpolar group exhibiting more extensive intrafilm hydrogen bonding and reduced interaction with the polar probe liquids. However, ρ_*NP*_ values do show a ∼10% decrease in nonpolar functional group exposure under water as the length increases from -ethyl to -butyl. These results suggest the ability of the longer and more flexible butyl chain to bury its nonpolar methyl group within the film, which contributes to an interface with a higher polar character than expected. These differences suggest that the simulation models may not fully capture the more complex surface behavior observed experimentally, such as subtle variations in local chain density, phase separation, or differences in surface packing or attachment.

### Determination of the Surface Free Energy of Amphiphilic Surfaces

The precision of the estimation of the surface free energy values depends on the number of probe liquids as well as the range covered by their chemical interactions, as quantified by the ratios of polar and dispersive energy components, γ_*LV*_^*P*^/γ_*LV*_^*D*^.^36,72^ In this study, we have selected glycerol, ethylene glycol, and thiodiglycol as the polar probe liquids for our model. Based on the MD simulation results, water disrupts the interchain hydrogen bonding to a higher degree than the other solvents, due to its small molecular size and high hydrogen bonding capacity with the polar functional groups, enhancing the responsive behavior beyond that seen for the other polar solvents studied. Therefore, water has not been included as a probe liquid in the model fitting described below. The γ_*LV*_^*P*^/γ_*LV*_^*D*^ ratio for the three remaining polar probe liquids spans from 1.1 to 0.4, with glycerol being the most polar and thiodiglycol the least. Diiodomethane, diiodopropane, and α-bromonaphthalene were chosen as the nonpolar probe liquids, each of which presents a surface tension sufficiently high to prevent complete wetting of the surface. As discussed in the Methods section (see eq 11), this model allows us to calculate both the polar contribution to the surface energy as a result of an enhanced exposure of polar groups (γ_*S*_2_ *V*_^*P*^) and also the group switchability (Δγ_*S*_2_ *V*–*S*_1_ *V*_ = γ_*S*_1_ *V*_ – γ_*S*_2_ *V*_) as a result of a variation of the surface composition, where negative values denote a greater switching response.

The surface free energies of the responsive amphiphilic monolayers are expected to depend on the length of the nonpolar group. The calculation of the switching surface energies of amphiphilic and single-component films using eqs 10 and 11 is provided in the Supporting Information, along with Owens-Wendt reference results (Figures S6–S11 and Tables S16–S19). These values align with others reported in the literature for surfaces exhibiting varying degrees of −OH and alkyl groups (Figure 8).^73^ These results show that the polar energy component rapidly decreases from a maximum at -H to a minimum at -ethyl, then increases and levels off for films with nonpolar terminal groups of -propyl, -isopropyl, and -butyl. This behavior highlights a distinct transition in interfacial composition that reflects how the molecular composition of the end group influences surface rearrangement.

**Figure 8:**
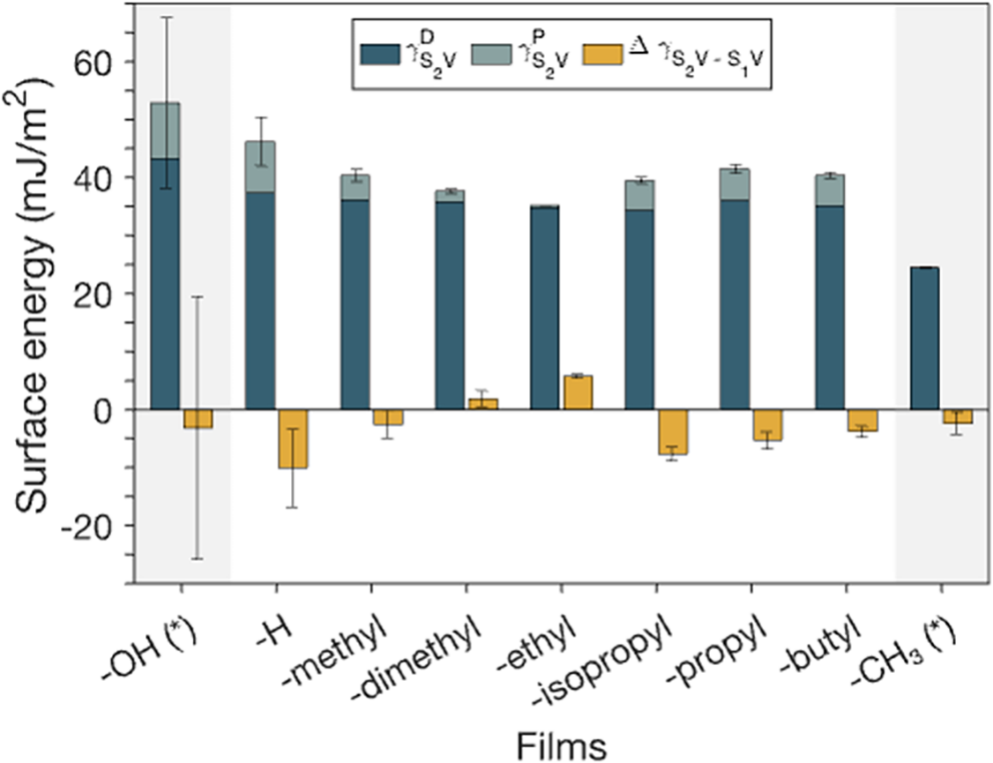
Surface free energies estimated using eq 11 on functionalized amphiphilic monolayers (white section) and purely polar and nonpolar monolayers (gray section). Polar and dispersive contributions to the total surface free energy (blue bars) of the area under a polar liquid (S_2_) are represented by γ_*S*_2_ *V*_^*P*^ and γ_*S*_2_ *V*_^*D*^, respectively. Δγ_*S*_2_V–*S*_1_V_ values (yellow bars) represent the difference in the surface free energies of the surfaces under air versus a polar liquid.

The initial decrease in the polar energy component suggests that increasing the nonpolar group length limits the accessibility of −OH groups to polar probe liquids. In the case of the -H film, the large γ_*S*_2_ *V*_^*P*^ value derived from low θ_*p*_ values (Figure 5) and the enhanced hydrogen bonding with polar liquids (Figure 7) indicate that polar groups are able to interact better with the polar liquids. Further, the SFG results (Figure 6b) that show a less structured –OH surface for this film support that the polar terminal group orients along the interface to expose both amide and some hydroxyl groups, while masking some of the underlying −CH_3_ functionalities from the backfill chains. For end groups with -methyl, -dimethyl, and -ethyl nonpolar groups, the dominant exposure of −CH_3_ and −CH_2_– functionalities suppresses polar interactions, as shown by the decrease in γ_*S*_2_ *V*_^*P*^ values. The minimum γ_*S*_2_ *V*_^*P*^ values obtained for these films also correspond with their calculated Δγ_*S*_2_ *V*–*S*_1_ *V*_ values, which approach zero or positive values. These results suggest that in these films with short nonpolar terminal functionalities, exposure to polar probe liquids does not significantly enhance the exposure of polar functionalities at the surface.

Films with end groups containing -isopropyl, -propyl, and -butyl functionalities show a sharp increase in the polar component of the S_2_ contacting area energy despite the increase in the hydrophobic characteristic of these functional groups. We attribute this reversal to the higher conformational freedom of longer alkyl functional groups, which may embed within the film, allowing the exposure of −OH groups, as suggested by the large −OH stretch signal in the SFG results for the film with the -butyl nonpolar functional group in Figure 6b. This increased flexibility re-establishes a polar character in these films as suggested by the plateau in γ_*S*_2_ *V*_^*P*^ values with longer nonpolar chains. Moreover, these same films also exhibit among the larger Δγ_*S*_2_ *V*–*S*_1_ *V*_ values, indicating pronounced switching behavior between air and polar liquids, an effect linked to enhanced molecular mobility and responsiveness.

A monolayer terminated solely with −OH groups displays very large error bars for the calculated energy components, which shows one of the limitations of a contact angle-based model to calculate the surface energy of purely polar surfaces, as shown elsewhere for similar films.^73^ The large error bar of the model on this polar surface arises from the poor fitting with a linear expression, an error that decreases in the Owens-Wendt calculation method since both polar and dispersive probe liquids are included in the same plot (Figure S6). In contrast, the CH_3_-terminated monolayer, serving as a fully nonpolar control, exhibits a γ_*S*_2_ *V*_^*P*^ value near zero, as expected. However, this film does show a small Δγ_*S*_2_ *V*–*S*_1_ *V*_ value of −2 mJ/m^2^, which is considered within the error in the fitting.

Analyzing the γ_*S*_2_ *L*_ values at the film–liquid interface provides deeper insights into the interfacial free energy minimization (Tables S20–S21). Figure 9 illustrates the γ_*S*_2_ *L*_ values estimated by using the γ_*S*_1_ *V*_ results obtained from eqs 11 and 5, as described in the Supporting Information. Consistent with contact angle data in Figure 5, the -H film exhibits the lowest γ_*S*_2_ *L*_ value with polar probe liquids (Figure 9a), indicating its ability to increase the interfacial polarity upon contact with a polar medium. Films with -methyl, -dimethyl, and -ethyl nonpolar functional groups show an interfacial energy that increases as the nonpolar composition of the end group increases, suggesting that these functionalities remain at the surface under these conditions, consistent with wetting (Figure 5) and surface energy (Figure 8) values. This trend can also be observed under nonpolar media (Figure 9b), where the increase in γ_*S*_2_ *L*_ values suggests that these surfaces are not able to minimize the interfacial energy with the dispersive media. Films with -isopropyl, -propyl, and -butyl groups exhibit an interfacial energy comparable to -H under polar liquids, despite the increase of nonpolar group size in these films (Figure 9a). This implies that longer nonpolar groups adopt conformations that expose polar functionalities in polar environments, lowering the interfacial free energy. Moreover, these films also show low γ_*S*_2_ *L*_ values under nonpolar media. In contrast to these, a purely nonpolar surface that also presents minimal γ_*S*_2_ *L*_ values under dispersive liquids does show large γ_*S*_2_ *L*_ values under polar media, indicating its inability to switch.

**Figure 9:**
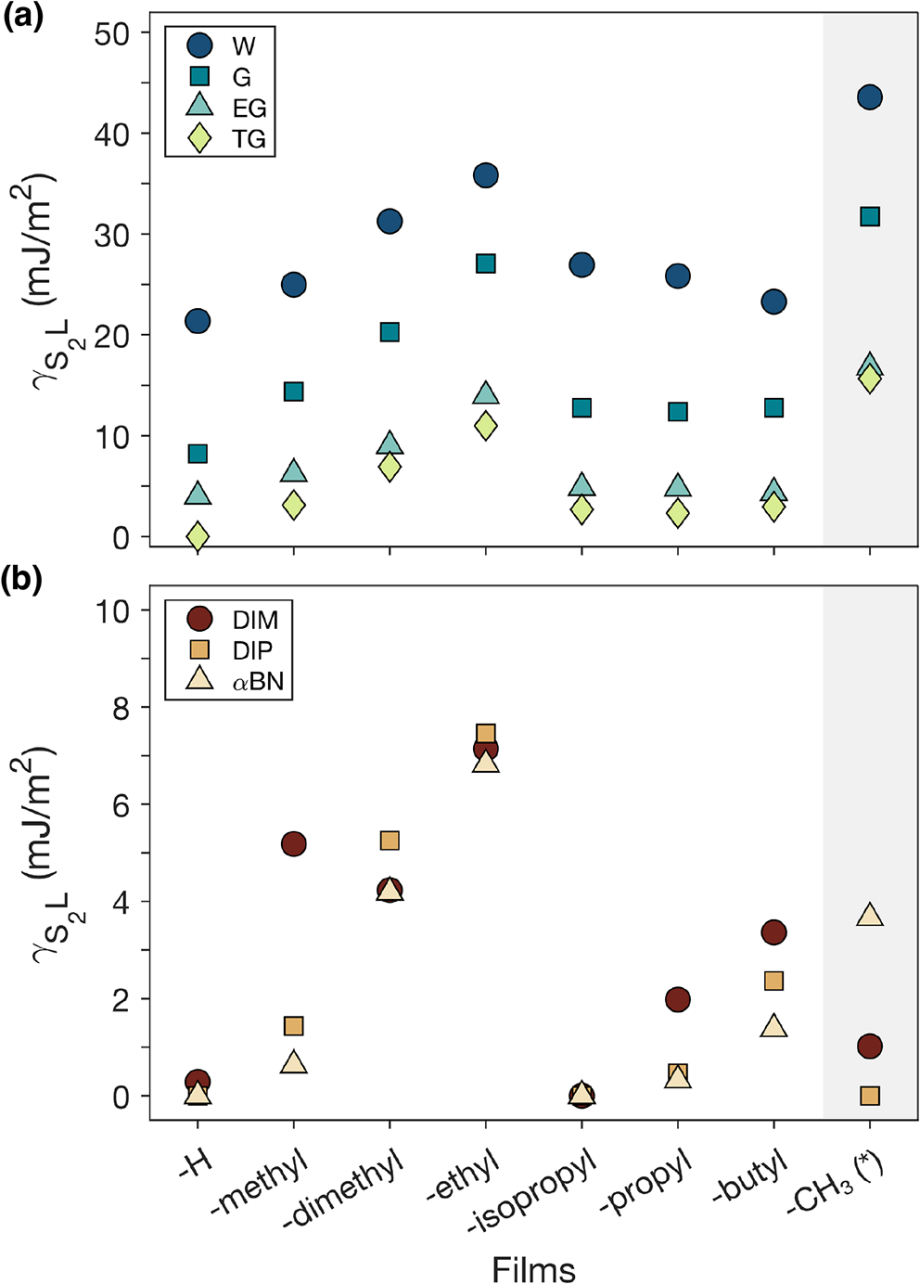
Interfacial free energies (γ_*S*_2_ *L*_) between the films and (a) polar and (b) dispersive probe liquids estimated using the model developed for responsive surfaces. Polar probe liquids are water (W), glycerol (G), ethylene glycol (EG), and thiodiglycol (TG). Dispersive probe liquids are diiodomethane (DIM), 1,3-diiodopropane (DIP), and α-bromonaphthalene (αBN).

## Conclusions

This work provides a detailed exploration of the design, characterization, and theoretical modeling of solvent-responsive amphiphilic monolayers, focusing on how end group composition influences interfacial behavior in response to environmental stimuli. By synthesizing a series of monolayers composed of polar and nonpolar functional groups of varying lengths, we demonstrated that such systems can undergo rapid and reversible surface reorganization when exposed to liquids of differing polarity. This interfacial adaptation occurs through molecular reorientation rather than large-scale structural rearrangement, which enables a fast response that is highly desirable for different applications. The results reveal that the length and structure of the functionalities that compose the end group critically influence the extent of surface rearrangement and thus the exposure of certain functional groups. In particular, monolayers bearing longer nonpolar groups exhibit enhanced conformational flexibility, enabling the burial of nonpolar groups and exposure of polar groups upon contact with polar media. The extent of this switching was evaluated using contact angle analysis, vibrational SFG spectroscopy, and MD simulations. Experimental observations from contact angle and SFG measurements align well with MD simulations, confirming the molecular basis of the observed responsiveness, although some discrepancies were observed in the predictive capacity of the simulations, especially when evaluating the differences in behavior of specific termini.

To further analyze and quantify this switching behavior, a surface energy calculation model was developed to incorporate responsive surface rearrangement into surface energy calculations, extending the Owens-Wendt framework to better describe adaptive interfacial systems. This model enables the estimation of both polar and dispersive contributions to the surface energy in different solvent environments, along with a new energy-based metric to describe the switching response of functional groups at the interface. Overall, the framework introduced here offers a robust methodology for assessing the switchability and surface energy of solvent-adaptive monolayers and potentially other switching thin films.

## Supplementary Material



## Notes

Contact angle data, fitting results for SFG data, MD simulations analysis of interchain and solvent-film hydrogen bonds, and solvation shell values for polar and nonpolar functional groups, fitting of contact angle data for the estimation of surface free energy values using both the Owens-Wendt equation and the model for responsive surfaces described in this work (PDF)

## Abbreviations

W: water
G: glycerol
EG: ethylene glycol
TG: thiodiglycol
DIM: diiodomethane
DIP: diiodopropane
αBN: α-bromonaphthalene
SFG: sum frequency generation
