# Supplementary material for: Evaluating the Structural Response of Amphiphilic Monolayers to Environmental Stimuli
Source: Langmuir. 2025 Sep 5;41(36):24906–17. doi: 10.1021/acs.langmuir.5c03473 (PMC12445002; doi:10.1021/acs.langmuir.5c03473)
Supplement: Supplementary file 1 [file la5c03473_si_001.pdf]

Supporting Information

Evaluating the Structural Response of Amphiphilic Monolayers to Environmental Stimuli

Allison V. Cordova-Huaman[1], Nicholas C. Craven[2], Marea J. Blake[3], Benjamin Doughty[3], Clare McCabe[1,4], and G. Kane Jennings[*,1]

(1) Department of Chemical and Biomolecular Engineering, Vanderbilt University, Nashville, TN 37235-1604, United States.

(2) Interdisciplinary Materials Science Graduate Program, Vanderbilt University, Nashville, TN 37235-1604, United States.

(3) Chemical Sciences Division, Oak Ridge National Laboratory, Oak Ridge, TN 37831, United States.

(4) School of Engineering and Physical Sciences, Heriot-Watt University, Scotland EH14 4AS, United Kingdom.

*Corresponding Author: G. Kane Jennings, kane.g.jennings@vanderbilt.edu

Section S1: Contact Angle Data

Table S1. Contact angles (deg) of single-component and amphiphilic monolayers with dispersive and polar probe liquids.

Film		Dispersive[a]			Polar[b]					
		$\theta_{s,\alpha BN}$	$\theta_{s,DIP}$	$\theta_{s,DIM}$	$\theta_{s,EG}$	$\theta_{s,TG}$	$\theta_{s,G}$	$\theta_{s,W}$	$\theta_{adv,W}$	$\theta_{rec,W}$
Single-component	C_{11}-OH	18 ± 1	19 ± 1	31 ± 1	30 ± 2	12 ± 2	49 ± 1	32 ± 5	37 ± 3	< 10
	C_{17}-CH_3	65 ± 1	61 ± 1	66 ± 1	84 ± 1	83 ± 1	99 ± 1	107 ± 1	115 ± 1	90 ± 1
Amphiphilic*	-H	30 ± 1	38 ± 1	45 ± 1	48 ± 1	48 ± 1	64 ± 1	78 ± 1	82 ± 1	35 ± 5
	-methyl	32 ± 1	39 ± 1	50 ± 1	49 ± 1	50 ± 1	69 ± 1	80 ± 2	83 ± 1	49 ± 1
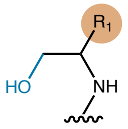	-dimethyl	36 ± 2	43 ± 3	46 ± 1	51 ± 2	53 ± 2	73 ± 1	84 ± 1	84 ± 3	47 ± 7
	-ethyl	39 ± 1	44 ± 1	48 ± 1	56 ± 1	56 ± 2	78 ± 3	86 ± 2	89 ± 1	54 ± 2
	-isopropyl	41 ± 1	45 ± 2	48 ± 2	56 ± 2	57 ± 2	73 ± 3	86 ± 1	88 ± 1	59 ± 1
	-propyl	35 ± 1	40 ± 1	48 ± 2	49 ± 1	51 ± 1	68 ± 1	82 ± 1	83 ± 1	49 ± 1
	-butyl	37 ± 2	43 ± 1	49 ± 2	48 ± 2	52 ± 1	68 ± 2	80 ± 2	82 ± 2	51 ± 2

[a,b] Probe liquids: α-Bromonaphthalene (αBN), diiodopropane (DIP), diiodomethane (DIM), thiodiglycol (TG), ethyleneglycol (EG), glycerol (G), and water (W).
* The nomenclature is based on the nonpolar functional group (R_1).

Section S2: Sum Frequency Generation (SFG) Spectroscopy

S2.1 SFG Spectra of Amphiphilic Monolayers without Backfilling Adsorbates

To probe how the polar and nonpolar functional groups in a monolayer film without free volume at the interface (i.e., monolayer without the backfilling CH_3-terminated adsorbates) restructure in response to polar (*e.g.*, water) and nonpolar (*e.g.,* air) environments, vibrational sum frequency generation spectroscopy (SFG) was employed. SFG is inherently selective to interfacial species and is capable of elucidating average orientation and associated local chemical environment. Figure S1 shows SFG spectra in the SSP polarization combination for monolayers composed of functionalized molecules with -H, -ethyl, and -butyl nonpolar functional groups exposed to air and water. This specific polarization combination and spectral region centered at ~3000 cm^{-1} reports on the symmetric CH stretches such as methylene (CH_2) and methyl (CH_3) groups. As can be seen in Figure S1a, a sharp peak is apparent at 2850 cm^{-1} in all three SSP spectra, which is attributed to the symmetric CH_2 stretch (CH_2-*ss*) present in the nonpolar region of these films at the film/air interface. A second, significantly weaker, shoulder is observed at 2870 cm^{-1} for the films with -ethyl and -butyl nonpolar functional groups, which is assigned to the symmetric CH_3 stretch (CH_3-*ss*) from the terminal CH_3 group on these functionalities. Noticeably, this peak is not seen for the film with an -H nonpolar group, since a CH_3 group is not present. Several broad peaks are observed at ~2905 cm^{-1}, ~2927 cm^{-1}, and ~2955 cm^{-1} attributed to overlapping CH_2 Fermi resonances (FRs) for the films with -H and -ethyl functional groups.[1,2] For the film with -butyl as the nonpolar group, four overlapping peaks are observed at ~2902 cm^{-1}, ~2917 cm^{-1}, ~2931 cm^{-1}, and ~2948 cm^{-1}, originating from a combination of FRs and out-of-phase asymmetric stretches from CH_2 and CH_3 groups. A lack of significant CH_3-*ss* signals indicates that the CH_3 groups are either highly disordered or lying parallel to the quartz substrate, without significant differences among the films.

When the monolayers are exposed to water, as shown in Figure S1b, two peaks are observed in all three spectra at 2845 cm^{-1} and 2901 cm^{-1}, corresponding to CH_2-*ss* and CH_2-FRs, respectively.[2] Remaining peaks on the high-frequency side are attributed to unresolved mixtures of FRs and out-of-phase asymmetric stretches, limiting a definitive assignment. Overall, the decrease in the CH_2-*ss* amplitude compared to the Fermi bands, in addition to the splitting of the FRs in comparison to the spectra from the films under air, indicates there is considerable reordering of the monolayers between the different solvent environments. Specifically, the disappearance of the CH_3-*ss* and CH_3-Fermi bands for the film with a -butyl nonpolar group suggests the hydrophobic functional group reorients into an azimuthally isotropic environment and/or the interface becomes more disordered.

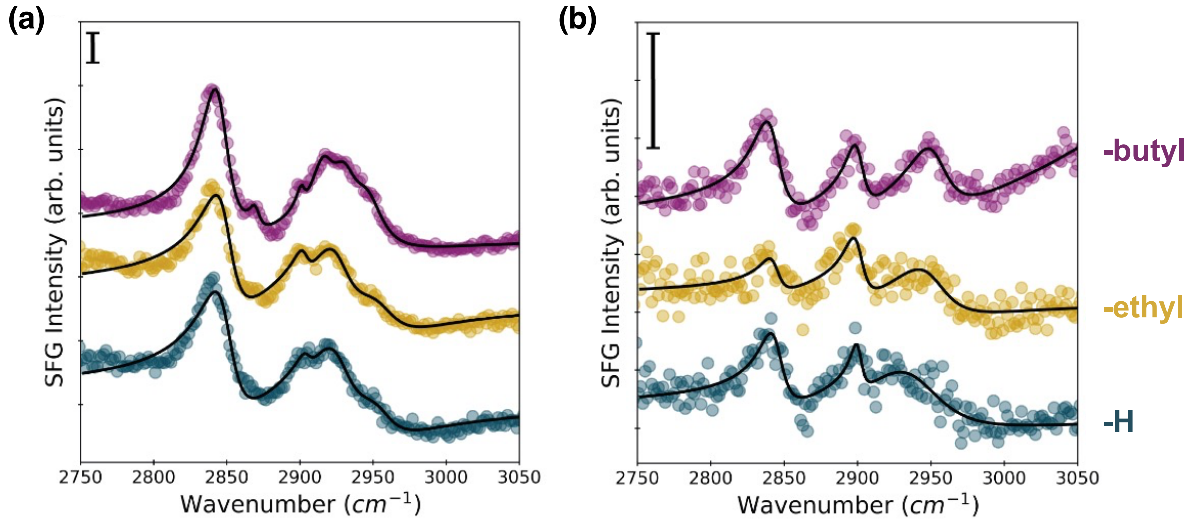

Figure S1. SFG spectra collected at SSP polarization from amphiphilic monolayers with -H, -ethyl, and -butyl nonpolar functional groups without backfilling adsorbates at the (a) film/air and (b) film/water interface. Data points are represented by dots, and the solid line represents the fit to the data. The scale bar shown for each plot equals 0.05 arbitrary units. Spectra are vertically offset for clarity.

S2.2 SFG Data Fitting

Table S2. Fitting results for SFG data obtained for amphiphilic monolayer samples with backfilling molecules with SSP polarization at the film/air interface at the CH spectral region (2750 – 3050 cm^{-1}). A_q and χ_{NR} are in arbitrary units, ω_q and Γ_q are in units of cm^{-1}, and Φ is in degrees.

Fitting Parameter	Film*		
	-H	-ethyl	-butyl
A_1	3.2 ± 0.3	3.2 ± 0.2	2.2 ± 0.2
ω_1	2844 ± 0.3	2843 ± 0.3	2843 ± 0.4
Γ_1	10.7 ± 0.7	9.8 ± 0.6	10.6 ± 0.8
A_2	2.4 ± 0.2	2.7 ± 0.2	1.8 ± 0.1
ω_2	2867 ± 0.3	2867 ± 0.3	2867 ± 0.3
Γ_2	8.9 ± 0.7	8.4 ± 0.5	8.5 ± 0.6
A_3	0.5 ± 0.1	0.3 ± 0.1	0.6 ± 0.2
ω_3	2903 ± 1	2905 ± 1	2907 ± 2
Γ_3	5.2 ± 1.2	4.7 ± 2.0	9.7 ± 3.3
A_4	7.0 ± 0.3	6.8 ± 0.3	4.0 ± 0.3
ω_4	2929 ± 0.4	2931 ± 0.4	2932 ± 0.5
Γ_4	17.1 ± 0.7	16.6 ± 0.7	15.2 ± 1.0
Φ	55 ± 2	60 ± 2	86 ± 2
χ_{NR}	-0.33 ± 0.004	-0.32 ± 0.004	-0.25 ± 0.004

* The nomenclature is based on the nonpolar functional group.

Table S3. Fitting results for SFG data obtained for amphiphilic monolayer samples with backfilling molecules with SSP polarization at the film/water interface at the CH spectral region (2750 – 3050 cm^{-1}). A_q and χ_{NR} are in arbitrary units, ω_q and Γ_q are in units of cm^{-1}, and Φ is in degrees.

Fitting Parameter	Film*		
	-H	-ethyl	-butyl
A_1	1.0 ± 0.2	1.2 ± 0.3	0.5 ± 0.2
ω_1	2840 ± 1	2840 ± 1	2839 ± 1
Γ_1	10.9 ± 1.7	10.9 ± 2.1	7.7 ± 1.9
A_2	0.8 ± 0.2	0.9 ± 0.3	1.2 ± 0.2
ω_2	2862 ± 1	2862 ± 1	2861 ± 1
Γ_2	9.5 ± 1.6	10.1 ± 2.5	11.9 ± 1.7
A_3	0.3 ± 0.1	0.7 ± 0.2	0.5 ± 0.1
ω_3	2900 ± 1	2903 ± 1	2900 ± 1
Γ_3	5.0 ± 1.1	9.3 ± 2.4	6.8 ± 1.2
A_4	1.4 ± 0.2	1.2 ±0.3	1.3 ± 0.2
ω_4	2925 ± 1	2924 ± 1	2924 ± 1
Γ_4	13.7 ± 1.6	10.4 ± 1.9	11.2 ± 1.3
A_5		0.4 ± 0.2	0.6 ± 0.3
ω_5		2956 ±3	2961 ± 2
Γ_5		9.6 ± 5.5	14.1 ± 5.0
A_6		8.2 ± 9.6	6.5 ± 4.4
ω_6		3138 ± 78	3069 ± 26
Γ_6		67.1 ± 32.0	53.9 ± 20.0
Φ	58 ± 4	61 ± 8	53 ± 12
χ_{NR}	-0.21 ± 0.002	-0.18 ± 0.02	-0.15 ± 0.02

* The nomenclature is based on the nonpolar functional group.

Table S4. Fitting results for SFG data obtained for amphiphilic monolayer samples without backfilling molecules with SSP polarization at the film/air interface at the CH spectral region (2750 – 3050 cm^{-1}). A_q and χ_{NR} are in arbitrary units, ω_q and Γ_q are in units of cm^{-1}, and Φ is in degrees.

Fitting Parameter	Film*		
	-H	-ethyl	-butyl
A_1	2.9 ± 0.1	2.4 ± 0.1	3.1 ± 0.1
ω_1	2850 ± 1	2850 ± 1	2846 ± 0.3
Γ_1	13.3 ± 0.6	12.1 ± 0.6	11.2 ± 0.5
A_2	0.2 ± 0.2	0.3 ± 0.1	0.3 ± 0.1
ω_2	2905 ± 2	2903 ± 1	2870 ± 1
Γ_2	5.8 ± 4.2	5.9 ± 2	4.6 ± 1.5
A_3	2.8 ± 0.8	2.2 ± 0.7	0.1 ± 0.1
ω_3	2927 ± 1	2927 ± 1	2902 ± 0.9
Γ_3	18.0 ± 3.7	16.4 ± 3.5	3.2 ± 1.8
A_4	1.4 ± 0.7	1.8 ± 0.6	1.0 ± 0.8
ω_4	2956 ± 3	2957 ± 3	2917 ± 2
Γ_4	17.6 ± 4.8	20.4 ± 4.5	9.5 ± 3.6
A_5			1.2 ± 1.5
ω_5			2931 ± 2
Γ_5			10.7 ± 6.9
A_6			1.9 ± 0.9
ω_6			2948 ± 3
Γ_6			14.4 ± 3.0
Φ	13 ± 2	14 ± 3	44 ± 2
χ_{NR}	-0.33 ± 0.002	-0.35 ± 0.002	-0.26 ± 0.003

* The nomenclature is based on the nonpolar functional group.

Table S5. Fitting results for SFG data obtained for amphiphilic monolayer samples without backfilling molecules with SSP polarization at the film/water interface at the CH spectral region (2750 – 3050 cm^{-1}). A_q and χ_{NR} are in arbitrary units, ω_q and Γ_q are in units of cm^{-1}, and Φ is in degrees.

Fitting Parameter	Film*		
	-H	-ethyl	-butyl
A_1	0.7 ± 0.1	0.3 ± 0.1	1.0 ± 0.1
ω_1	2845 ± 1	2844 ± 2	2843 ± 1
Γ_1	9.8 ± 1.5	7.6 ± 2.9	11.1 ± 1.1
A_2	0.2 ± 0.1	0.4 ± 0.1	0.5 ± 0.1
ω_2	2901 ± 1	2901 ± 1	2901 ± 1
Γ_2	4.7 ± 1.6	7.8 ± 1.8	7.5 ± 1.2
A_3	2.3 ± 0.3	1.1 ± 0.2	1.1 ± 0.2
ω_3	2941 ± 3	2952 ± 3	2954 ± 1
Γ_3	34.8 ± 4.6	22.6 ± 4.3	16.6 ± 2.2
A_4			13.0 ± 4.0
ω_4			3121 ± 14
Γ_4			81.7 ± 14.0
Φ	36 ± 5	31 ± 8	37 ± 9
χ_{NR}	-0.18 ± 0.002	-0.19 ± 0.002	-0.13 ± 0.01

* The nomenclature is based on the nonpolar functional group.

Table S6. Values from fitting results for amphiphilic monolayer samples with backfilling molecules with SSP polarization at the film/water interface at the stitched regions (2750 – 3200 cm^{-1}). Phase was set constant at $\Phi = 0°$. A_q and χ_{NR} are in arbitrary units and ω_q and Γ_q are in units of cm^{-1}.

Fitting Parameter	Film*		
	-H	-ethyl	-butyl
A_1	0.5 ± 0.2	0.6 ± 0.2	0.3 ± 0.2
ω_1	2847 ± 2	2844 ± 1	2843 ± 2
Γ_1	8.8 ± 3.1	7.4 ± 2.1	7.7 ± 4.4
A_2	1.3 ± 0.3	1.7 ± 0.4	1.4 ± 0.3
ω_2	2867 ± 1	2868 ± 2	2864 ± 2
Γ_2	11.3 ± 2.1	15.3 ± 3.1	13.9 ± 2.7
A_3	0.3 ± 0.1	0.28 ± 0.2	0.4 ± 0.1
ω_3	2903 ± 1	2903 ± 2	2901 ± 1
Γ_3	5.3 ± 2.1	6.3 ± 3.6	6.1 ± 2.1
A_4	1.7 ± 0.6	1.5 ± 0.7	1.8 ± 0.3
ω_4	2928 ± 2	2926 ± 2	2925 ± 1
Γ_4	18.8 ± 2.9	14.6 ± 4.9	14.1 ± 2.5
A_5	2.4 ± 0.8	2.7 ± 0.9	1.2 ± 0.3
ω_5	2962 ± 6	2958 ± 6	2962 ± 2
Γ_5	28.3 ± 7.0	28.4 ± 6.1	17.6 ± 3.8
A_6	35.9 ± 7.3	59.3 ± 5.0	64.9 ± 2.8
ω_6	3292 ± 17	3261 ± 6	3235 ± 2
Γ_6	140.7 ± 21.4	143.4 ± 10.1	120.9 ± 4.8
χ_{NR}	-0.15 ± 0.02	-0.10 ± 0.01	-0.06 ± 0.01

* The nomenclature is based on the nonpolar functional group.

Section S3: Molecular Dynamics (MD) Simulation Analysis

Figure S2 and Table S7 show the hydrogen bonds that form between the solvent atoms and the films. Water and ethylene glycol both show the most film-solvent interaction, with the strongest hydrogen bonding occurring with the film with -H as the nonpolar functional group. Generally, we observe that the films with short nonpolar functional groups form fewer hydrogen bonds within the film (i.e., interchain hydrogen bonds) than with the solvent (Tables S8-9).

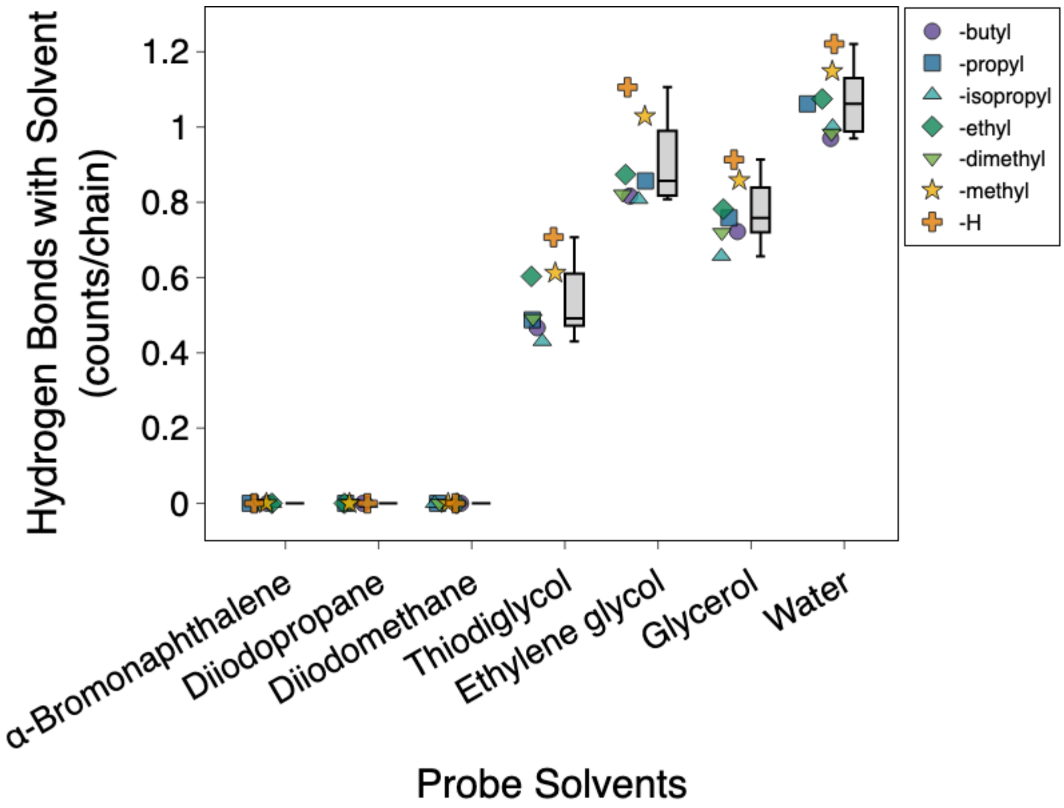

Figure S2. Hydrogen bonds between the polar functional group in the amphiphilic film and the solvent. All nonpolar solvents show no hydrogen bonding to the film, whereas polar solvents do form hydrogen bonds with the hydroxyl terminal group. Each solvent includes a neighboring boxplot of all seven films at that state, with mean, 25th percentile, 75th percentile, minimum, and maximum values displayed.

Table S7. Number of interchain hydrogen bonds in amphiphilic films under air and dispersive media (counts/chain).

Films*	Air			Dispersive media								
				α-Bromonaphthalene			Diiodopropane			Diiodomethane		
	Mean	Std	95% CI	Mean	Std	95% CI	Mean	Std	95% CI	Mean	Std	95% CI
-H	0.86	0.08	0.10	0.85	0.04	0.04	0.83	0.02	0.02	0.82	0.03	0.04
-methyl	0.78	0.03	0.03	0.76	0.04	0.05	0.72	0.02	0.02	0.74	0.03	0.03
-dimethyl	0.74	0.00	0.00	0.71	0.05	0.06	0.60	0.09	0.10	0.68	0.05	0.06
-ethyl	0.78	0.03	0.03	0.81	0.02	0.02	0.79	0.04	0.05	0.75	0.04	0.04
-isopropyl	0.73	0.00	0.00	0.73	0.02	0.03	0.76	0.02	0.02	0.78	0.00	0.00
-propyl	0.78	0.01	0.02	0.80	0.01	0.01	0.76	0.06	0.07	0.81	0.06	0.07
-butyl	0.77	0.03	0.04	0.78	0.03	0.03	0.78	0.02	0.02	0.81	0.03	0.03

* The nomenclature is based on the nonpolar functional group of the amphiphilic terminal groups.

Table S8. Number of interchain hydrogen bonds in amphiphilic films under polar media (counts/chain).

Films*	Polar media											
	Thiodiglycol			Ethylene glycol			Glycerol			Water		
	Mean	Std	95% CI	Mean	Std	95% CI	Mean	Std	95% CI	Mean	Std	95% CI
-H	0.32	0.05	0.05	0.20	0.01	0.01	0.26	0.04	0.04	0.14	0.01	0.01
-methyl	0.34	0.03	0.04	0.23	0.02	0.02	0.27	0.02	0.02	0.14	0.00	0.01
-dimethyl	0.32	0.08	0.10	0.27	0.03	0.04	0.27	0.03	0.03	0.16	0.02	0.02
-ethyl	0.32	0.01	0.02	0.34	0.01	0.02	0.35	0.02	0.02	0.18	0.04	0.05
-isopropyl	0.43	0.01	0.01	0.32	0.01	0.01	0.36	0.04	0.05	0.22	0.03	0.03
-propyl	0.42	0.05	0.06	0.34	0.01	0.02	0.37	0.04	0.05	0.20	0.01	0.02
-butyl	0.44	0.03	0.04	0.38	0.05	0.06	0.41	0.04	0.04	0.27	0.05	0.05

* The nomenclature is based on the nonpolar functional group of the amphiphilic terminal groups.

Table S9. Number of hydrogen bonds between the polar functional group in amphiphilic films and the polar media (counts/chain).

Films*	Polar media											
	Thiodiglycol			Ethylene glycol			Glycerol			Water		
	Mean	Std	95% CI	Mean	Std	95% CI	Mean	Std	95% CI	Mean	Std	95% CI
-H	0.71	0.03	0.04	1.11	0.01	0.01	0.91	0.05	0.06	1.22	0.02	0.03
-methyl	0.61	0.03	0.04	1.03	0.00	0.01	0.86	0.02	0.02	1.15	0.03	0.03
-dimethyl	0.49	0.06	0.07	0.82	0.01	0.01	0.72	0.02	0.03	0.98	0.02	0.02
-ethyl	0.60	0.01	0.01	0.87	0.04	0.04	0.78	0.02	0.02	1.07	0.05	0.05
-isopropyl	0.43	0.01	0.01	0.81	0.02	0.03	0.66	0.03	0.04	1.00	0.04	0.05
-propyl	0.49	0.06	0.07	0.86	0.05	0.06	0.76	0.03	0.03	1.06	0.04	0.04
-butyl	0.47	0.01	0.01	0.82	0.06	0.07	0.72	0.01	0.01	0.97	0.07	0.08

* The nomenclature is based on the nonpolar functional group of the amphiphilic terminal groups.

Solvation shell values of polar (ρ_P) and nonpolar (ρ_{NP}) groups are provided in Figures S3 and S4, respectively. This parameter allows us to evaluate the interactions between the polar and nonpolar functional groups and the probe liquids. If the solvent interactions are stronger with the polar functional groups, then the ρ_P values are higher and the surface of the film exhibits a more polar character. Conversely, when interactions are greater with the nonpolar functional groups, ρ_{NP} values increase, and the interface is more nonpolar. The probe liquids appear at different levels on the plot based in part on their solvent density.

Tables S10–13 present the solvation shell values necessary to assess the switching behavior of the films exposed to polar and nonpolar probe solvents. For instance, the film functionalized with a -butyl nonpolar group presents a ρ_P in α-bromonaphthalene (αBN) of 18.7 atoms/nm³, while in water it increases to 38 atoms/nm³. The corresponding ρ_{NP} values for this film are 31.9 and 22.6 atoms/nm³ under αBN and water, respectively. The larger ρ_{NP} value under αBN suggests a predominantly nonpolar character under dispersive media, while the increase in ρ_P value upon exposure to water, depicts the increase of polar functionalities under polar media, which aligns with the experimental contact angle measurements for this film with -butyl as the nonpolar functional group.

Figure S5 illustrates the polar character of the films across all probe liquids. In contact with nonpolar solvents, the films exhibit a polar character below 50%, indicating a preferential exposure of nonpolar functional groups at the interface. In contrast, exposure to polar probe liquids leads to a clear increase in polar character, with average values above 50%. This increase in polarity is most pronounced in water, where the polar character reaches 60%. Complete values are listed in Tables S14-15.

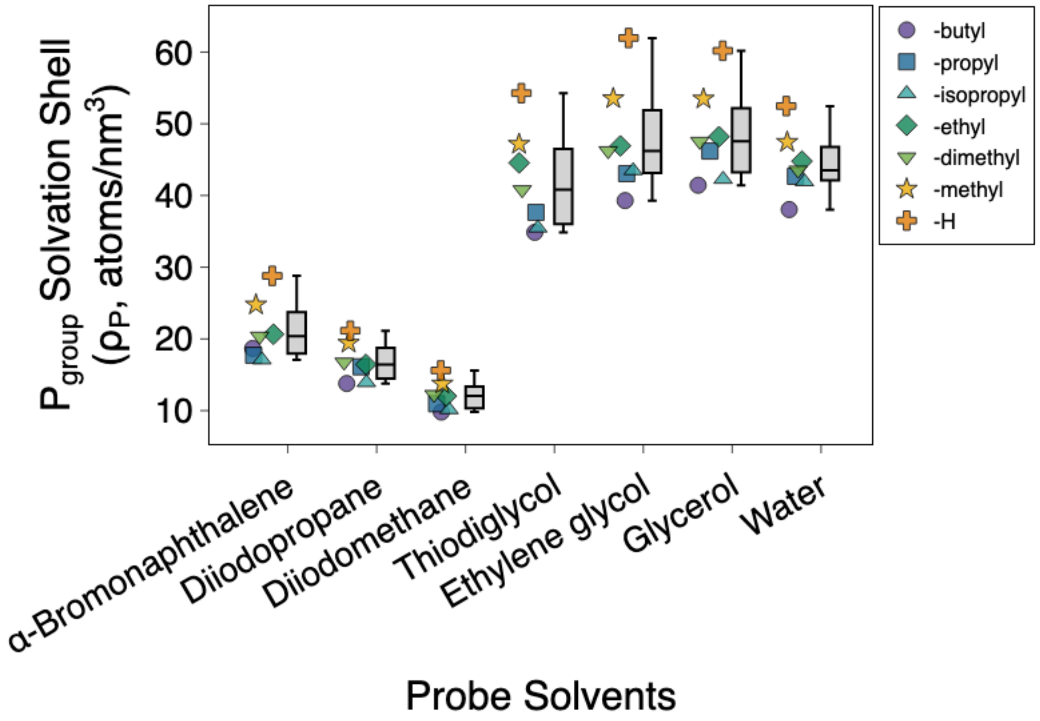

Figure S3. Solvation shell of polar groups (ρ_P) in amphiphilic films under dispersive and polar liquids. Solvation shell is the number of solvent atoms (hydrogens included) within a 0.5 nm radial shell of the oxygen atom in the hydroxyl group of the polar functional group.

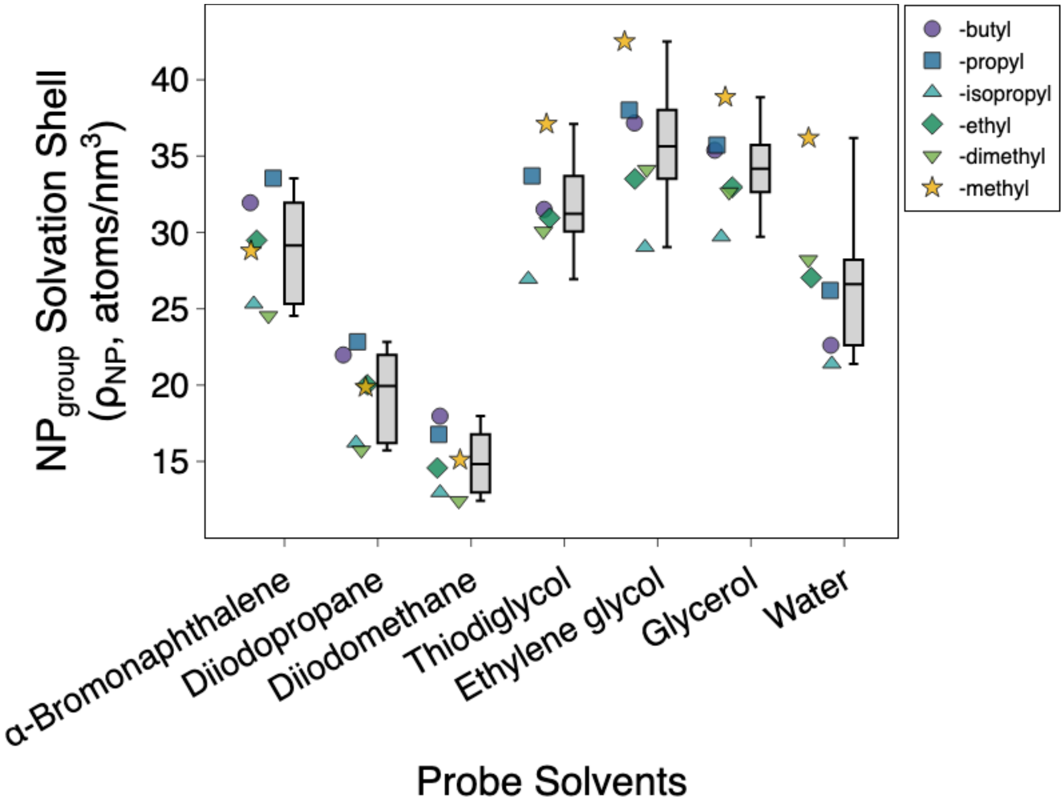

Figure S4. Solvation shell of nonpolar groups (ρ_{NP}) in amphiphilic films under dispersive and polar liquids. Solvation shell is the number of solvent atoms (hydrogens included) within a 0.5 nm radial shell of the carbon atom in the last methyl group of the nonpolar functional group. The -H film is not shown because there is no terminal methyl group to use for the calculation. However, the values for the CH_2 hydrogens as the nonpolar terminal atom can be found in Tables S12 and S13.

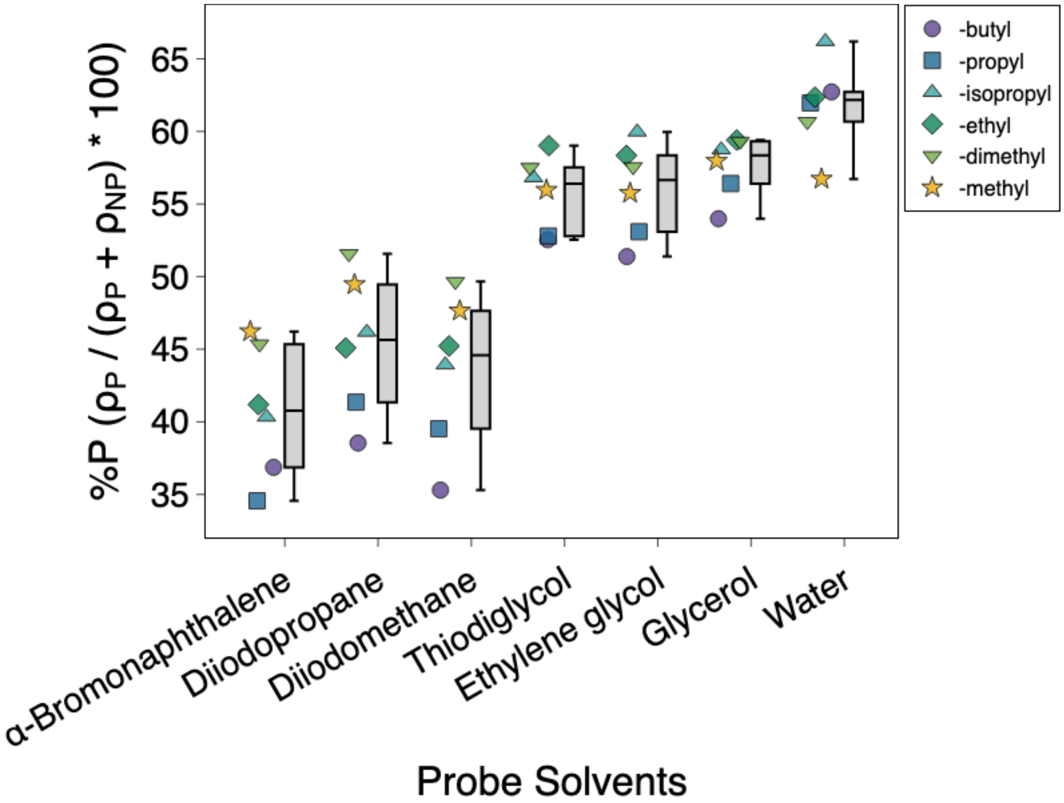

Figure S5. Contribution of polar functional groups to the polar character of the interface (%P) of amphiphilic films under dispersive and polar liquids. Solvation shell ρ_{group} is the number of solvent atoms (hydrogens included) within a 0.5 nm radial shell of the oxygen atom in the hydroxyl group of the polar functional group, and the methyl carbon in the nonpolar functional group. The polar and nonpolar contributions are then weighted to form %P. Each solvent includes a neighboring boxplot of all seven films at that state, with mean, 25th percentile, 75th percentile, minimum, and maximum values displayed. The -H film is not shown because there is no terminal methyl to use for the calculation, however, the values for the CH_2 hydrogens as the nonpolar terminal atom can be found in Tables S12 and S13.

Table S10. Solvation shell values of the polar functional group (ρ_P) in amphiphilic films under dispersive media (atoms/nm^3).

Films*	Dispersive media								
	α-Bromonaphthalene			Diiodopropane			Diiodomethane		
	Mean	Std	95% CI	Mean	Std	95% CI	Mean	Std	95% CI
-H	28.8	0.2	0.2	21.1	0.7	0.7	15.6	0.1	0.2
-methyl	24.8	1.4	1.6	19.4	0.3	0.4	13.7	0.3	0.3
-dimethyl	20.4	0.6	0.7	16.8	0.8	0.9	12.2	0.1	0.1
-ethyl	20.7	1.6	1.8	16.4	0.4	0.4	12.0	0.3	0.3
-isopropyl	17.1	0.5	0.6	13.9	0.9	1.0	10.1	0.4	0.5
-propyl	17.7	0.2	0.2	16.1	1.4	1.5	11.0	0.3	0.4
-butyl	18.7	1.2	1.3	13.8	0.4	0.4	9.8	0.3	0.3

* The nomenclature is based on the nonpolar functional group of the amphiphilic terminal groups.

Table S11. Solvation shell values of the polar functional group (ρ_P) in amphiphilic films under polar media (atoms/nm^3).

Films*	Polar media											
	Thiodiglycol			Ethylene glycol			Glycerol			Water		
	Mean	Std	95% CI	Mean	Std	95% CI	Mean	Std	95% CI	Mean	Std	95% CI
-H	54.3	1.9	2.2	62.0	1.0	1.1	60.2	2.2	2.5	52.5	1.2	1.4
-methyl	47.2	1.6	1.8	53.5	0.5	0.5	53.5	0.8	0.9	47.4	1.2	1.3
-dimethyl	40.8	2.8	3.2	46.2	0.4	0.5	47.5	0.4	0.5	43.5	1.1	1.3
-ethyl	44.5	0.2	0.3	46.9	0.7	0.8	48.2	0.5	0.6	44.8	1.6	1.9
-isopropyl	35.5	1.0	1.2	43.5	0.5	0.6	42.3	1.3	1.5	41.9	2.0	2.3
-propyl	37.6	1.8	2.1	43.0	3.2	3.6	46.2	1.9	2.2	42.7	1.6	1.8
-butyl	34.9	1.1	1.3	39.3	1.2	1.4	41.4	0.7	0.8	38.0	2.0	2.3

* The nomenclature is based on the nonpolar functional group of the amphiphilic terminal groups.

Table S12. Solvation shell values of the nonpolar functional group (ρ_{NP}) in amphiphilic films under dispersive media (atoms/nm^3).

Films*	Dispersive media								
	α-Bromonaphthalene			Diiodopropane			Diiodomethane		
	Mean	Std	95% CI	Mean	Std	95% CI	Mean	Std	95% CI
-H	28.3	1.0	1.1	18.8	1.3	1.5	14.0	0.6	0.7
-methyl	28.8	0.9	1.0	19.9	0.6	0.7	15.1	0.5	0.5
-dimethyl	24.5	0.8	0.9	15.7	0.8	0.9	12.4	0.4	0.5
-ethyl	29.5	1.5	1.7	20.0	0.5	0.6	14.6	0.5	0.6
-isopropyl	25.3	1.6	1.8	16.2	0.7	0.8	13.0	0.9	1.0
-propyl	33.6	0.7	0.7	22.8	1.2	1.3	16.8	0.3	0.4
-butyl	31.9	0.6	0.7	22.0	0.4	0.5	18.0	0.7	0.8

* The nomenclature is based on the nonpolar functional group of the amphiphilic terminal groups.

Table S13. Solvation shell values of the nonpolar functional groups (ρ_{NP}) in amphiphilic films under polar media (atoms/nm^3).

Films*	Polar media											
	Thiodiglycol			Ethylene glycol			Glycerol			Water		
	Mean	Std	95% CI	Mean	Std	95% CI	Mean	Std	95% CI	Mean	Std	95% CI
-H	40.4	0.8	0.9	46.5	1.5	1.8	43.7	1.9	2.1	38.9	1.9	2.1
-methyl	37.1	0.5	0.5	42.5	2.1	2.4	38.9	3.5	4.0	36.2	0.3	0.3
-dimethyl	30.1	0.3	0.4	34.1	2.3	2.7	32.6	2.2	2.5	28.2	0.9	1.1
-ethyl	30.9	0.9	1.0	33.5	1.5	1.7	33.0	1.7	2.0	27.0	1.0	1.1
-isopropyl	26.9	0.4	0.4	29.0	1.1	1.2	29.7	2.0	2.2	21.4	0.8	0.9
-propyl	33.7	2.9	3.2	38.0	2.9	3.3	35.7	2.8	3.2	26.2	1.0	1.1
-butyl	31.5	1.9	2.1	37.2	0.4	0.4	35.4	3.1	3.5	22.6	1.8	2.0

* The nomenclature is based on the nonpolar functional group of the amphiphilic terminal groups.

Table S14. Weighted percentage of polar groups (%P) calculated from ρ_{NP} and ρ_P in amphiphilic films under dispersive media.

Films*	Dispersive media								
	α-Bromonaphthalene			Diiodopropane			Diiodomethane		
	Mean	Std	95% CI	Mean	Std	95% CI	Mean	Std	95% CI
-H	46.2	2.1	2.3	49.5	1.1	1.3	47.6	1.3	1.5
-methyl	45.4	0.6	0.7	51.6	2.2	2.5	49.7	0.7	0.8
-dimethyl	41.2	3.1	3.5	45.1	1.1	1.3	45.2	0.4	0.4
-ethyl	40.3	2.3	2.6	46.2	1.8	2.1	43.9	2.8	3.1
-isopropyl	34.6	0.6	0.7	41.4	2.9	3.3	39.5	1.2	1.3
-propyl	36.9	1.9	2.1	38.5	1.0	1.2	35.3	1.1	1.2
-butyl	46.2	2.1	2.3	49.5	1.1	1.3	47.6	1.3	1.5

Table S15. Weighted percentage of polar groups (%P) calculated from ρ_{NP} and ρ_P in amphiphilic films under polar media.

Films*	Polar media											
	Thiodiglycol			Ethylene glycol			Glycerol			Water		
	Mean	Std	95% CI	Mean	Std	95% CI	Mean	Std	95% CI	Mean	Std	95% CI
-H	56.0	0.5	0.6	55.8	1.4	1.6	58.0	2.6	2.9	56.7	0.5	0.6
-methyl	57.5	2.0	2.2	57.6	1.9	2.2	59.3	1.8	2.0	60.7	1.4	1.6
-dimethyl	59.0	0.8	0.9	58.3	0.7	0.8	59.4	1.5	1.7	62.4	1.2	1.3
-ethyl	56.8	1.0	1.1	60.0	0.9	1.0	58.7	2.3	2.6	66.2	1.8	2.1
-isopropyl	52.8	0.9	1.0	53.1	3.5	4.0	56.4	2.9	3.3	62.0	0.4	0.5
-propyl	52.5	2.1	2.4	51.4	0.8	1.0	54.0	1.9	2.2	62.7	2.5	2.8
-butyl	56.0	0.5	0.6	55.8	1.4	1.6	58.0	2.6	2.9	56.7	0.5	0.6

Section S3: Estimation of Surface Free Energy (SFE) Components

S3.1 SFE Calculated by the Owens-Wendt Method

The surface free energy of the functionalized samples was determined using the Owens-Wendt model, which considers that the total surface free energy (γ_{SV}) is composed of a polar (γ_{SV}^{P}) and a dispersive (γ_{SV}^{D}) component:

$$\gamma_{SV} = \gamma_{SV}^{P} + \gamma_{SV}^{D} \quad \text{(Eq. S1)}$$

The Owens-Wendt equation can be expressed in a linearized form for solid-liquids systems, enabling calculation of these components using experimental values of the contact angles (Table S1) and liquid surface tension, γ_{LV} (Table 1) obtained from the films and the probe liquids, respectively:

$$\frac{\gamma_{LV}(\cos\theta + 1)}{2\sqrt{\gamma_{LV}^{D}}} = \sqrt{\frac{\gamma_{LV}^{P}}{\gamma_{LV}^{D}}} \cdot \sqrt{\gamma_{SV}^{P}} + \sqrt{\gamma_{SV}^{D}} \quad \text{(Eq. S2)}$$

This Eq. S2 can be further simplified as:

$$\frac{\gamma_{LV}(\cos\theta + 1)}{2\sqrt{\gamma_{LV}^{D}}} = \sqrt{\frac{\gamma_{LV}^{P}}{\gamma_{LV}^{D}}} \cdot m_1 + b_1 \quad \text{(Eq. S3)}$$

In this linear form, the intercept (b_1) corresponds to the square root of the dispersive component of the surface energy ($\sqrt{\gamma_{SV}^{D}}$), and the slope (m_1) corresponds to the square root of the polar component ($\sqrt{\gamma_{SV}^{P}}$).

We applied the Owens-Wendt equation to calculate the surface energy of films terminated with -OH and -CH_3 terminal groups (Figure S6), as well as amphiphilic films (Figure S7). The linear fits of our experimental data are summarized in Table S16, and the corresponding calculated surface energies and their components are presented in Table S17.

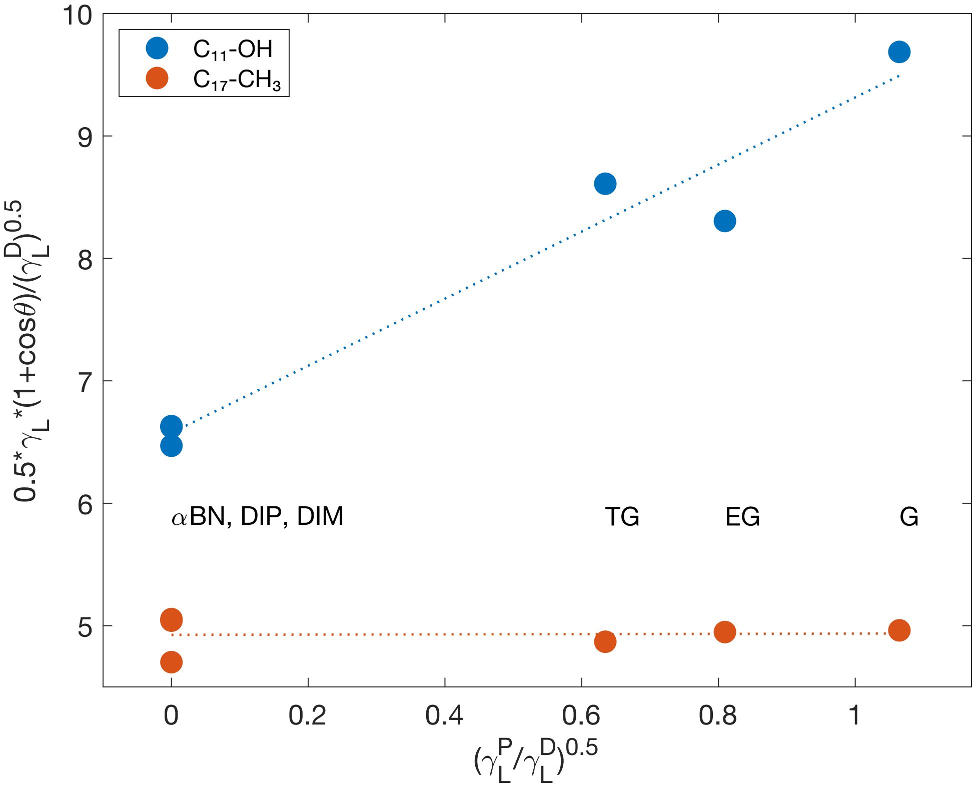

Figure S6. Owens-Wendt plot of films with only -OH and $-CH_3$ terminal groups using contact angle data with six probe liquids (α-bromonaphthalene (αBN), diiodopropane (DIP), diiodomethane (DIM), thiodiglycol (TG), ethylene glycol (EG), and glycerol (G)).

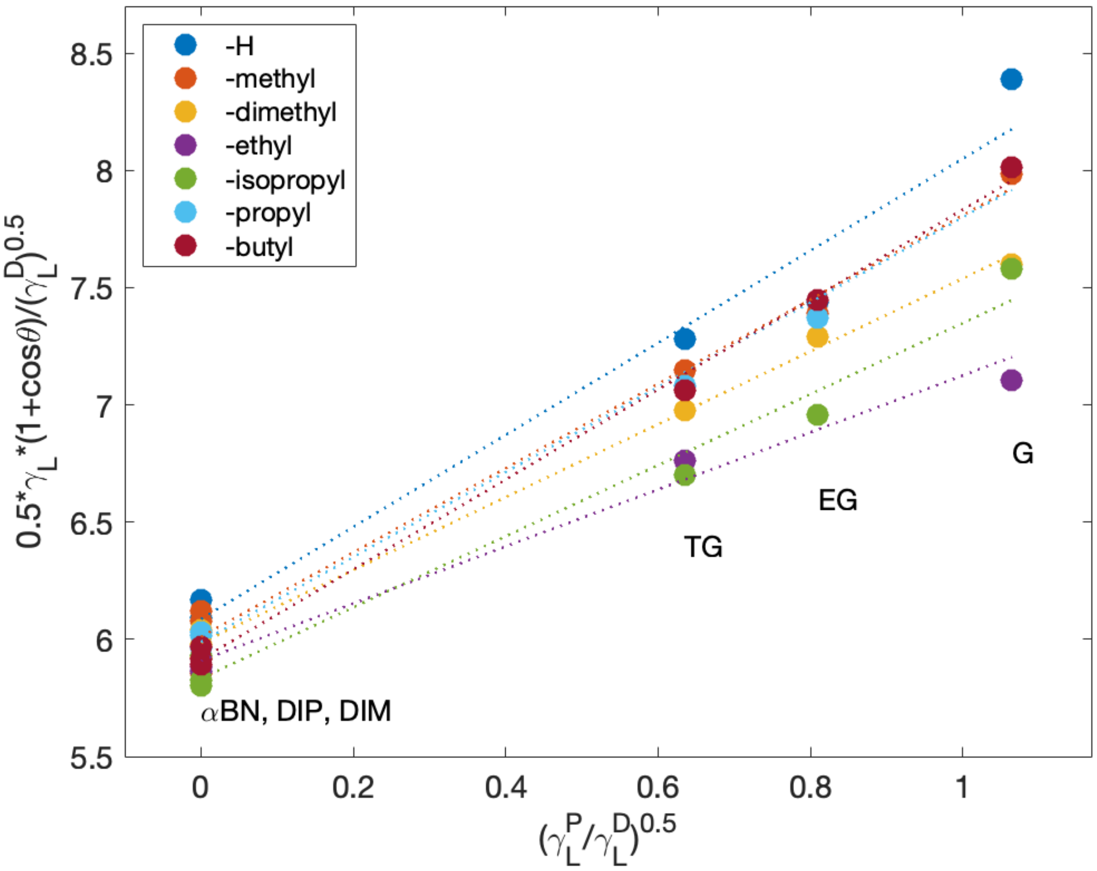

Figure S7. Owens-Wendt plot of amphiphilic films using contact angle data with six probe liquids (α-bromonaphthalene (αBN), diiodopropane (DIP), diiodomethane (DIM), thiodiglycol (TG), ethylene glycol (EG), and glycerol (G))

Table S16. Fitting results of the experimental contact angle data from single-component and amphiphilic films to the Owens-Wendt equation.

Film		m_1	b_1
Single-component	C_{11}-OH	2.7 ± 0.3	6.6 ± 0.2
	C_{17}-CH_3	0.0 ± 0.1	4.93 ± 0.08
Amphiphilic*	-H	2.0 ± 0.2	6.1 ± 0.1
	-methyl	1.8 ± 0.1	6.0 ± 0.1
	-dimethyl	1.55 ± 0.05	5.98 ± 0.03
	-ethyl	1.2 ± 0.1	5.91 ± 0.04
	-isopropyl	1.5 ± 0.1	5.8 ± 0.1
	-propyl	1.8 ± 0.1	5.98 ± 0.04
	-butyl	1.9 ± 0.1	5.91 ± 0.03

* The nomenclature is based on the nonpolar functional group.

Table S17. Surface free energy components of single-component and amphiphilic films calculated from the fitting of the contact angle data to the Owens-Wendt equation (mJ/m^2).

Film		γ_{SV}^{D}	γ_{SV}^{P}	γ_{SV}
Single-component	C_{11}-OH	43.2 ± 2.3	7.5 ± 1.6	50.7 ± 2.8
	C_{17}-CH_3	24.2 ± 0.8	0.000 ± 0.003	24.3 ± 0.8
Amphiphilic*	-H	37.0 ± 1.1	3.9 ± 0.6	40.9 ± 1.3
	-methyl	36.1 ± 0.8	3.2 ± 0.4	39.4 ± 0.8
	-dimethyl	35.8 ± 0.4	2.4 ± 0.2	38.2 ± 0.4
	-ethyl	34.9 ± 0.5	1.5 ± 0.2	36.4 ± 0.5
	-isopropyl	34.0 ± 0.7	2.3 ± 0.3	36.3 ± 0.8
	-propyl	35.9 ± 0.5	3.3 ± 0.3	39.1 ± 0.6
	-butyl	35.0 ± 0.4	3.7 ± 0.2	38.7 ± 0.4

* The nomenclature is based on the nonpolar functional group.

S3.2 SFE Calculated using the Model for Responsive Surfaces

As described in the main text, the surface free energy or responsive surfaces can also be evaluated using a two-step method that separates the contributions from dispersive and polar probe liquids. This approach is based on the following expressions for dispersive (Eq. S4) and polar (Eq. S5) liquids.

$$\gamma_{LV}(1+\cos\theta_d) = 2\sqrt{\gamma_{LV}^{D}}\cdot\sqrt{\gamma_{S_2V}^{D}} \quad \text{(Eq. S4)}$$

$$\gamma_{LV}(1+\cos\theta_p) - 2\sqrt{\gamma_{S_2V}^{D}\cdot\gamma_{LV}^{D}} = \Delta\gamma_{S_2V-S_1V} + 2\sqrt{\gamma_{LV}^{P}}\cdot\sqrt{\gamma_{S_2V}^{P}} \quad \text{(Eq. S5)}$$

These equations can be expressed in a linearized form as:

$$\gamma_{LV}(1+\cos\theta_d) = 2\sqrt{\gamma_{LV}^{D}}\cdot m_2 \quad \text{(Eq. S6)}$$

$$\gamma_{LV}(1+\cos\theta_p) - 2\sqrt{\gamma_{S_2V}^{D}\cdot\gamma_{LV}^{D}} = b_3 + 2\sqrt{\gamma_{LV}^{P}}\cdot m_3 \quad \text{(Eq. S7)}$$

The dispersive component of the surface energy in contact with the probe liquid ($\gamma_{S_2V}^{D}$) is obtained from Eq. S6 by plotting the left-hand side versus $2\sqrt{\gamma_{LV}^{D}}$, which corresponds to the slope of this fit (m_2). Similarly, Eq. S5 enables the determination of the polar component ($\gamma_{S_2V}^{P}$) and the variation of the surface energy upon exposure to a polar probe liquid ($\Delta\gamma_{S_2-S_1}$). The fitting of these equations for single-component and amphiphilic films is shown in Figures S8-11, as well as the surface energy components, which are reported in Tables S18 and S19.

This two-step method provides a detailed breakdown of surface energy contributions, allowing for separate determination of dispersive and polar components, the variation of the surface energy upon exposure to new environments, and the total interfacial energies of the film under liquids, as defined in Eq. S8 and S9:

$$\gamma_{S_2V} = \gamma_{S_2V}^{D} + \gamma_{S_2V}^{D} \quad \text{(Eq. S8)}$$

$$\gamma_{S_1V} = \Delta\gamma_{S_2V-S_1V} + \gamma_{S_2V} \quad \text{(Eq. S9)}$$

The calculated interfacial energy values based on this model are presented in Table S19.

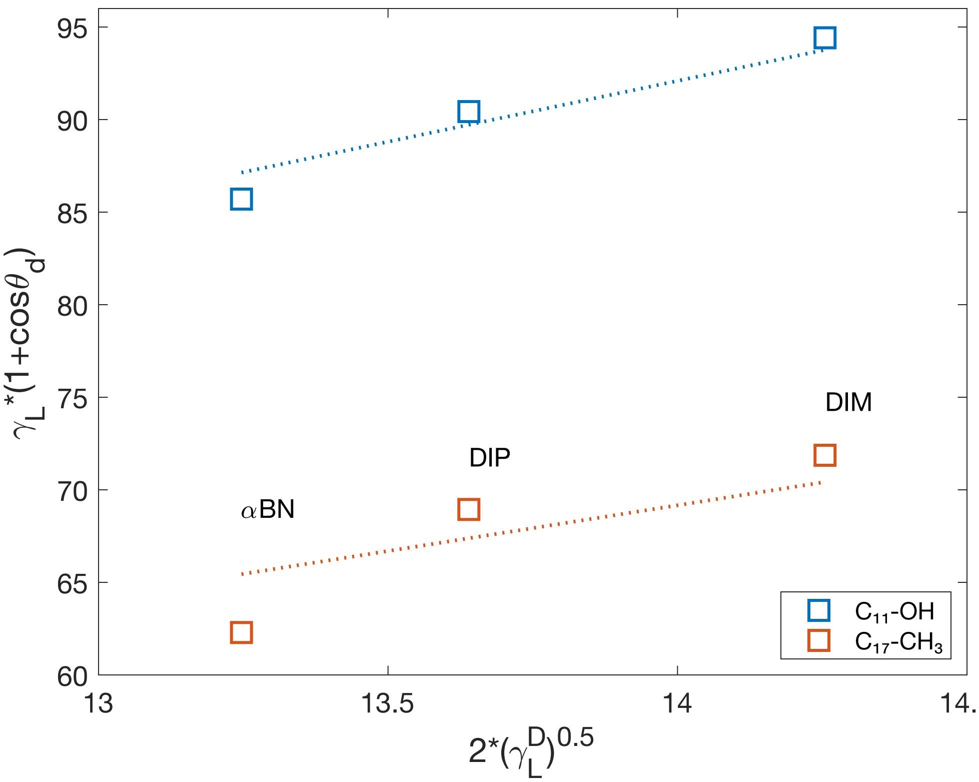

Figure S8. Calculation of surface free energies of single-component films with only -OH and $-CH_3$ terminal groups in contact with dispersive probe liquids (α-bromonaphthalene (αBN), diiodopropane (DIP), and diiodomethane (DIM)) using Eq. S6.

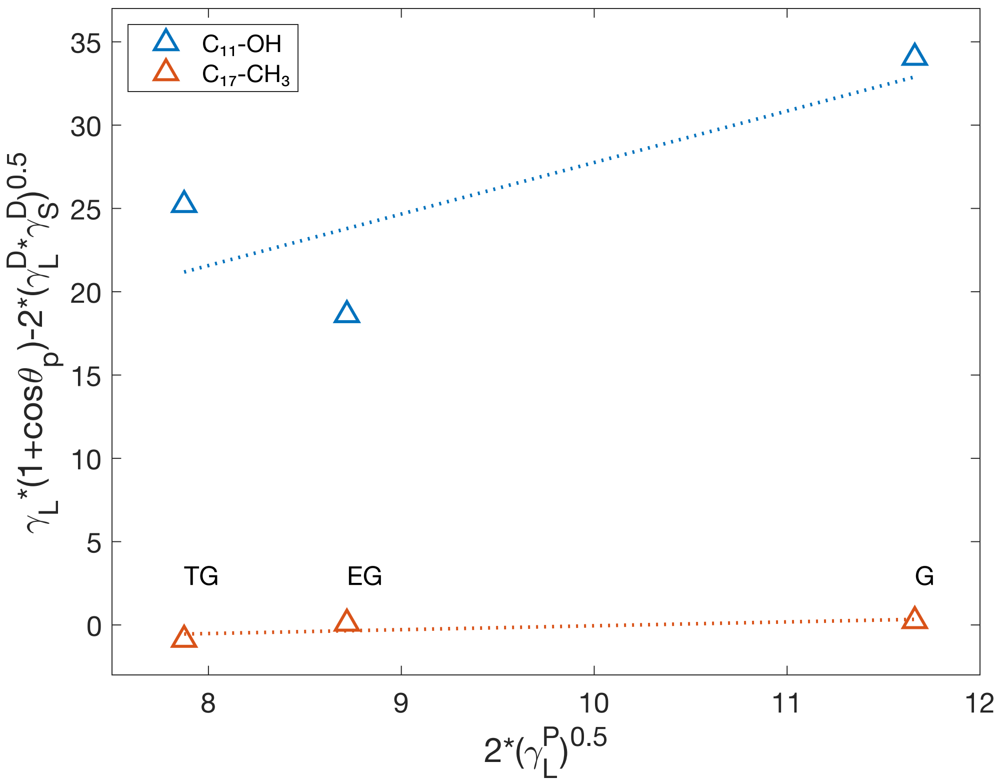

Figure S9. Calculation of surface free energies of single-component films with only -OH and $-CH_3$ terminal groups in contact with polar probe liquids (thiodiglycol (TG), ethylene glycol (EG), and glycerol (G)) using Eq. S7.

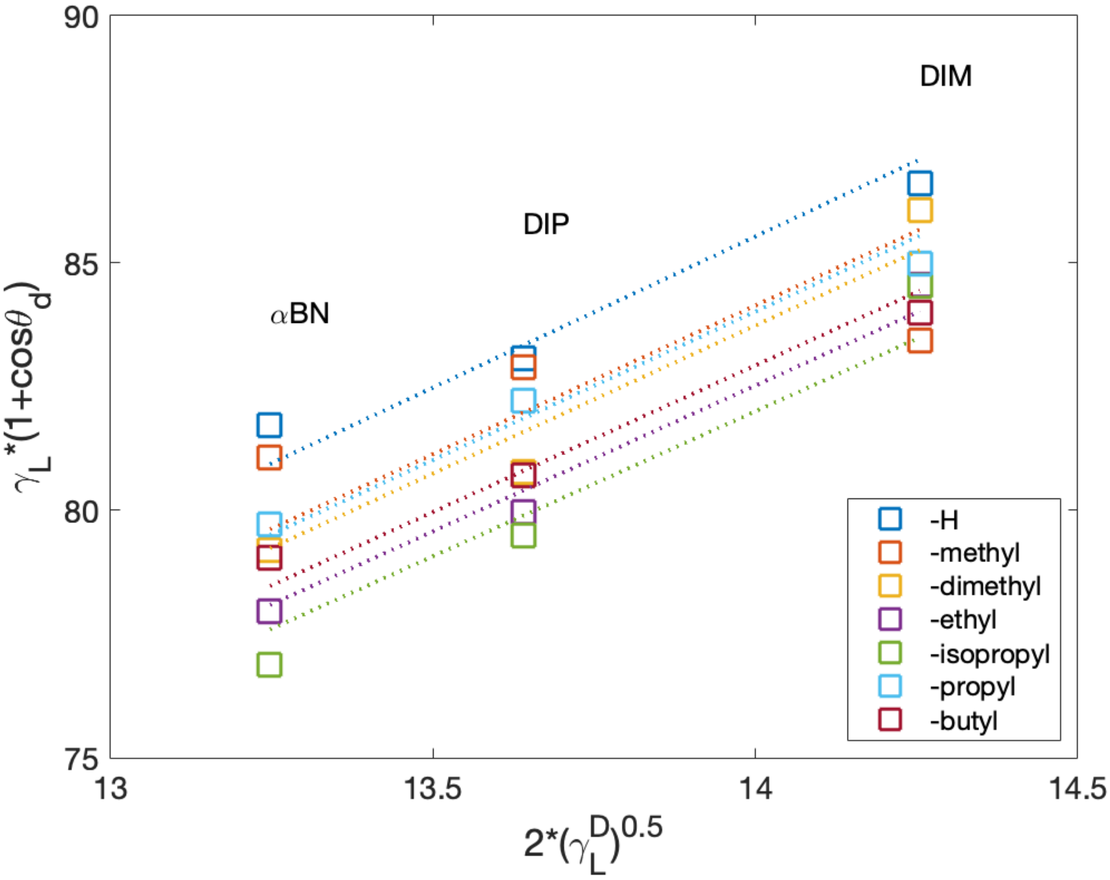

Figure S10. Calculation of surface free energies of amphiphilic films in contact with dispersive probe liquids (α-bromonaphthalene (αBN), diiodopropane (DIP), and diiodomethane (DIM)) using Eq. S6.

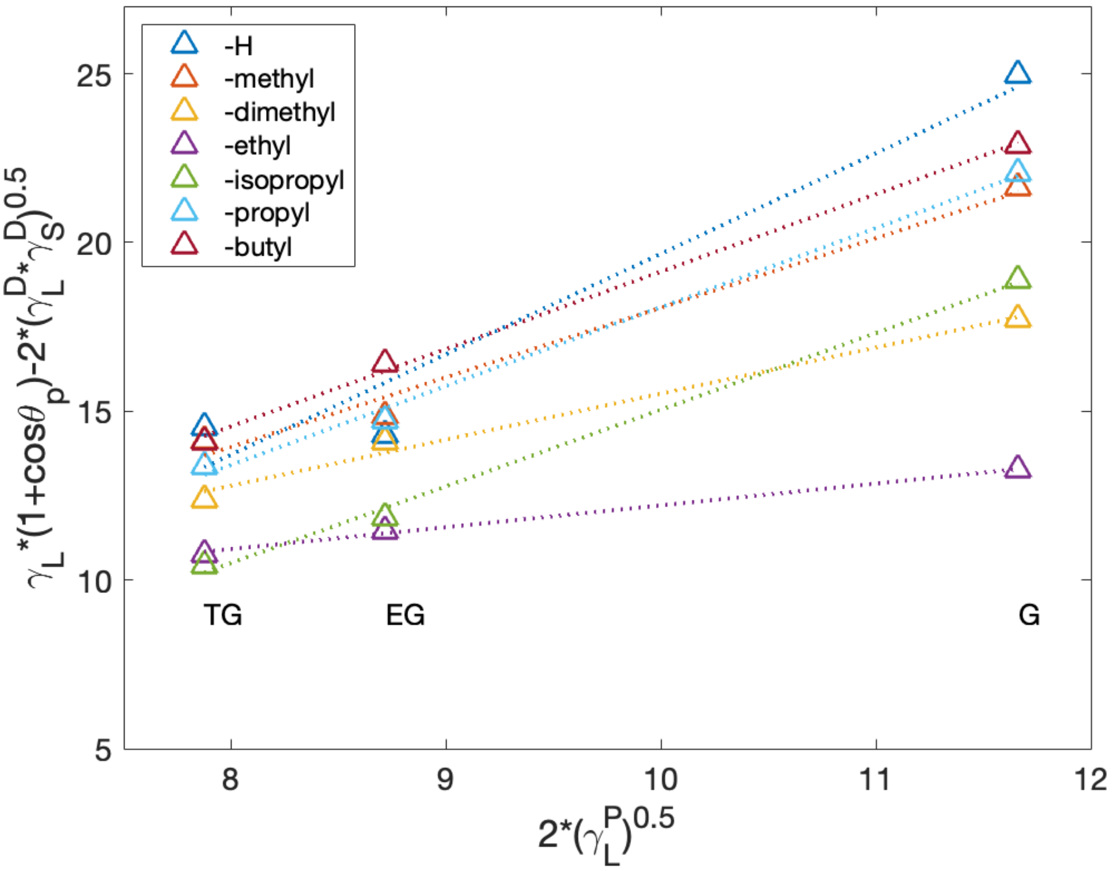

Figure S11. Calculation of surface free energies of amphiphilic films in contact with polar probe liquids (thiodiglycol (TG), ethylene glycol (EG), and glycerol (G)) using Eq. S7.

Table S18. Fitting results of the experimental contact angle data from single-component and amphiphilic films to Eq. S6 and S7.

Film		m_2	m_3	b_3
Single-component	C_{11}-OH	6.58 ± 0.05	3.1 ± 2.4	-3.2 ± 22.7
	C_{17}-CH_3	4.9 ± 0.1	0.2 ± 0.2	-2.4 ± 1.9
Amphiphilic*	-H	6.11 ± 0.03	3.0 ± 0.7	-10.1 ± 6.7
	-methyl	6.01 ± 0.08	2.1 ± 0.3	-2.5 ± 2.5
	-dimethyl	5.98 ± 0.03	1.4 ± 0.1	1.9 ± 1.4
	-ethyl	5.89 ± 0.02	0.65 ± 0.04	5.7 ± 0.4
	-isopropyl	5.86 ± 0.04	2.3 ± 0.1	-7.7 ± 1.2
	-propyl	6.00 ± 0.02	2.3 ± 0.2	-5.3 ± 1.5
	-butyl	5.92 ± 0.02	2.3 ± 0.1	-3.8 ± 0.9

* The nomenclature is based on the nonpolar functional group.

Table S19. Surface free energy components of single-component and amphiphilic films calculated from the fitting of the contact angle data to Eq. S6 and S7 (mJ/m^2).

Film		$\gamma^{D}_{S_2V}$	$\gamma^{P}_{S_2V}$	γ_{S_2V}	$\Delta\gamma_{S_2V-S_1V}$	γ_{S_1V}
Single-component	C_{11}-OH	43.3 ± 0.7	9.6 ± 14.7	52.8 ± 14.7	-3.2 ± 22.7	49.7 ± 27.0
	C_{17}-CH_3	24.4 ± 1.1	0.05 ± 0.09	24.5 ± 1.1	-2.4 ± 1.9	22.1 ± 2.2
Amphiphilic*	-H	37.3 ± 0.4	8.9 ± 4.2	46.2 ± 4.2	-10.1 ± 6.7	36.1 ± 7.9
	-methyl	36.1 ± 1.0	4.2 ± 1.1	40.4 ± 1.5	-2.5 ± 2.5	37.8 ± 2.9
	-dimethyl	35.8 ± 0.4	1.9 ± 0.4	37.6 ± 0.6	1.9 ± 1.4	39.5 ± 1.5
	-ethyl	34.7 ± 0.2	0.4 ± 0.1	35.2 ± 0.3	5.7 ± 0.4	40.9 ± 0.4
	-isopropyl	34.3 ± 0.5	5.2 ± 0.6	39.5 ± 0.7	-7.7 ± 1.2	31.8 ± 1.4
	-propyl	36.0 ± 0.2	5.5 ± 0.7	41.5 ± 0.8	-5.3 ± 1.5	36.2 ± 1.7
	-butyl	35.1 ± 0.3	5.3 ± 0.4	40.3 ± 0.5	-3.8 ± 0.9	36.6 ± 1.1

* The nomenclature is based on the nonpolar functional group.

S3.3 Calculation of γ_{SL} components

The interfacial free energy (γ_{S_2L}) between the functionalized films and the probe liquids can be estimated using the energy balance on a sessile droplet, based on the surface energy of the film in air (γ_{S_1V}) on Table S19 and cosθ values in Table S1:

$$\gamma_{S_2L} = \gamma_{S_1V} - \gamma_{LV} \cos\theta \quad \text{(Eq. S10)}$$

The calculated γ_{S_2L} values for both dispersive and polar probe liquids are summarized in Tables S20 and S21, respectively. Due to the limited accuracy of this model when applied to highly polar surfaces, γ_{S_2L} values were only calculated for amphiphilic and nonpolar surfaces.

Table S20. Interfacial free energy (γ_{S_2L}) component of single-component and amphiphilic films under dispersive probe liquids (mJ/m^2).

Film		$\gamma_{S_2L}^{\alpha BN}$	$\gamma_{S_2L}^{DIP}$	$\gamma_{S_2L}^{DIM}$
Single-component	C_{17}-CH_3	4 ± 2	0 †	1 ± 2
Amphiphilic*	-H	0 †	0 †	0 ± 8
	-methyl	0.6 ± 2.9	1.4 ± 2.9	5 ± 3
	-dimethyl	4.2 ± 1.8	5.3 ± 2.1	4.2 ± 1.8
	-ethyl	6.8 ± 0.8	7.5 ± 0.7	7.1 ± 0.8
	-isopropyl	0.0 ± 1.6	0.0 ± 1.7	0.0 ± 2.1
	-propyl	0.3 ± 1.8	0.5 ± 1.7	2.0 ± 2.1
	-butyl	1.4 ± 1.3	2.4 ± 1.3	3.4 ± 1.8

* The nomenclature is based on the nonpolar functional group.
† Negative results for γ_{SL} values have been considered as 0 mJ/m^2.

Table S21. Interfacial free energy (γ_{S_2L}) component of single-component and amphiphilic films under polar probe liquids (mJ/m^2).

Film		$\gamma_{S_2L}^{EG}$	$\gamma_{S_2L}^{TG}$	$\gamma_{S_2L}^{G}$	$\gamma_{S_2L}^{W}$
Single-component	C_{17}-CH_3	17 ± 2	16 ± 2	32 ± 2	44 ± 2
Amphiphilic*	-H	4 ± 8	0 ± 8	8 ± 8	21 ± 8
	-methyl	6 ± 3	3 ± 3	14 ± 3	25 ± 4
	-dimethyl	9 ± 2	7 ± 2	20.3 ± 1.9	31 ± 2
	-ethyl	14.0 ± 0.9	11.0 ± 1.5	27 ± 3	36 ± 2
	-isopropyl	4.9 ± 1.9	3 ± 2	13 ± 3	26.9 ± 1.9
	-propyl	4.8 ± 1.8	2.3 ± 1.9	12 ± 2	25 ± 2
	-butyl	4.3 ± 1.5	2.9 ± 1.5	13 ± 2	23 ± 2

* The nomenclature is based on the nonpolar functional group.

References

(1) Lu, R.; Gan, W.; Wu, B.; Zhang, Z.; Guo, Y.; Wang, H. C−H Stretching Vibrations of Methyl, Methylene and Methine Groups at the Vapor/Alcohol (n = 1−8) Interfaces. *J. Phys. Chem. B* **2005**, *109* (29), 14118–14129. https://doi.org/10.1021/jp051565q.

(2) Wang, H.; Gan, W.; Lu, R.; Rao, Y.; Wu, B. Quantitative Spectral and Orientational Analysis in Surface Sum Frequency Generation Vibrational Spectroscopy (SFG-VS). *Int. Rev. Phys. Chem.* **2005**, *24* (2), 191–256. https://doi.org/10.1080/01442350500225894.
